# Experimental Toxoplasmosis in Rats Induced Orally with Eleven Strains of *Toxoplasma gondii* of Seven Genotypes: Tissue Tropism, Tissue Cyst Size, Neural Lesions, Tissue Cyst Rupture without Reactivation, and Ocular Lesions

**DOI:** 10.1371/journal.pone.0156255

**Published:** 2016-05-26

**Authors:** Jitender P. Dubey, Leandra R. Ferreira, Mohammad Alsaad, Shiv K. Verma, Derron A. Alves, Gary N. Holland, Glenn A. McConkey

**Affiliations:** 1 United States Department of Agriculture, Agricultural Research Service, Beltsville Agricultural Research Center, Animal Parasitic Diseases Laboratory, Beltsville, Maryland, United States of America; 2 Faculty of Biological Sciences, University of Leeds, Miall Building, Clarendon Way, Leeds, United Kingdom; 3 Veterinary Pathology Services Joint Pathology Center, 606 Stephen Sitter Ave. Silver Spring, Maryland, United States of America; 4 Ocular Inflammatory Disease Center, University of California at Los Angles Stein Eye Institute and Department of Ophthalmology, David Geffen School of Medicine at UCLA, Los Angeles, California, United States of America; University of Wisconsin Medical School, UNITED STATES

## Abstract

**Background:**

The protozoan parasite *Toxoplasma gondii* is one of the most widely distributed and successful parasites. *Toxoplasma gondii* alters rodent behavior such that infected rodents reverse their fear of cat odor, and indeed are attracted rather than repelled by feline urine. The location of the parasite encysted in the brain may influence this behavior. However, most studies are based on the highly susceptible rodent, the mouse.

**Methodology/Principal Findings:**

Latent toxoplasmosis was induced in rats (10 rats per *T*. *gondii* strains) of the same age, strain, and sex, after oral inoculation with oocysts (natural route and natural stage of infection) of 11 *T*. *gondii* strains of seven genotypes. Rats were euthanized at two months post inoculation (p.i.) to investigate whether the parasite genotype affects the distribution, location, tissue cyst size, or lesions. Tissue cysts were enumerated in different regions of the brains, both in histological sections as well in saline homogenates. Tissue cysts were found in all regions of the brain. The tissue cyst density in different brain regions varied extensively between rats with many regions highly infected in some animals. Overall, the colliculus was most highly infected although there was a large amount of variability. The cerebral cortex, thalamus, and cerebellum had higher tissue cyst densities and two strains exhibited tropism for the colliculus and olfactory bulb. Histologically, lesions were confined to the brain and eyes. Tissue cyst rupture was frequent with no clear evidence for reactivation of tachyzoites. Ocular lesions were found in 23 (25%) of 92 rat eyes at two months p.i. The predominant lesion was focal inflammation in the retina. Tissue cysts were seen in the sclera of one and in the optic nerve of two rats. The choroid was not affected. Only tissue cysts, not active tachyzoite infections, were detected. Tissue cysts were seen in histological sections of tongue of 20 rats but not in myocardium and leg muscle.

**Conclusion/Significance:**

This study reevaluated in depth the rat model of toxoplasmosis visualizing cyst rupture and clarified many aspects of the biology of the parasite useful for future investigations.

## Introduction

*Toxoplasma gondii* is one of the most well studied parasites because of its medical and veterinary importance, ease of *in vitro* cultivation (ability to grow in many cell lines), being readily recognizable by light microscopy, ease of genetic manipulation, and availability of different isolates with varying virulence [1]. It is a coccidian parasite with felids as its definitive host, and all warm blooded animals, including humans as intermediate hosts. *Toxoplasma gondii* infections are widely prevalent in humans and animals worldwide [1]. People become infected postnatally mainly by ingesting tissue cysts from undercooked meat, or consuming food or water contaminated with oocysts. However, only a small percentage of exposed adult humans develop clinical symptoms following exposure. It is unknown whether the severity of toxoplasmosis in immunocompetent persons is due to the parasite strain, host variability, or to other factors. Attention has been focused on the genetic variability among *T*. *gondii* isolates from apparently healthy and sick hosts [2]. Severe cases of toxoplasmosis have been reported in immunocompetent patients in association with atypical *T*. *gondii* genotypes in certain countries [2–6]. Historically, *T*. *gondii* was considered to be clonal with low genetic diversity and grouped into three types: I, II, and III [7]. However, recent studies have revealed a greater genetic diversity of *T*. *gondii*, particularly in isolates from South America [8,9].

A variant of type II (NE-II) *T*. *gondii* strain was found associated with prematurity and severe disease at birth in congenitally infected children in the USA [10]. Little is known of the association of genotype and clinical disease in animals [1]. Type II strains are the most prevalent in Europe and the United States [11].

After a few multiplication cycles, *T*. *gondii* tachyzoites convert into bradyzoites that encyst in many organs, predominantly in brain and muscle [12,13]. It is thought that most hosts remain infected for life and have persistent tissue cysts [1]. It is presumed that tissue cysts rupture from time to time but in an immunocompetent host the bradyzoites released from tissue cysts are killed by host immune factors, mostly by cell-mediated immunity. However, in an immunosuppressed host, the released bradyzoites can transform into tachyzoites, and reactivate acute infection. The reactivation can be clinically disastrous. Many patients with Acquired Immune Deficiency Syndrome (AIDS) die of toxoplasmosis or have severe vision loss from persistent, active retinal infection [14]. How and when tissue cysts rupture is unknown. However, administration of exogenous corticosteroids in large doses can facilitate proliferation and development of clinical lesions if there is reactivation (for unknown reasons) but does not cause the reactivation itself [1]. It is thought that congenitally–infected children, especially infected in the first two trimesters, although born asymptomatic, at any age can develop clinical toxoplasmosis. Ocular toxoplasmosis has been diagnosed in patients that were born asymptomatic two decades previously [1,15].

Most information on the biology of tissue cysts has been derived from studies in rodents, principally mice (Tables 1 and 2). Mice are a convenient model because of the availability of many different strains, and knockouts for specific traits. However, there are two main drawbacks of the mouse model. Although laboratory mice are highly susceptible to infection, parasitemia and congenital infection can occur during chronic infection in the absence of exogenous infection [16–18]. Additionally, all strains of mice die (unless medicated) when infected with type I genotype strains of *T*. *gondii*, irrespective of the dose. Compared with type I, types II and III are relatively avirulent for mice; however, oocysts of most strains are pathogenic for mice [19]. Currently, there are many genotypes of *T*. *gondii* with varying degrees of virulence to mice. Rats, in contrast, are more resistant to clinical toxoplasmosis, and simulate *T*. *gondii* infection in humans. However, comparatively, little is known of the biology of *T*. *gondii* infection in rats and many older studies were performed with *T*. *gondii* strains that had been serially passaged in the laboratory that may have altered their phenotypic traits [1,20].

**Table 1:** Summary of studies concerning biology of tissue cysts in rodents.

Rodent					*Toxoplasma gondii*				Cyst biology		References
Host	Strain	Sex	Age	N=	Strain genotype	Route	Dose	Duration	Distribution	Method	
Rat	Long-Evans	M	8 w	6	II, Prugniaud^a^	i.p.	1 million tachyzoites		More cysts in amygdalar region	Histology-data pooled, bioluminescence	[21]
Rat	Wistar	M	NS	28	I, RH^b^	i.p	100 or 1000 tachyzoites	25 days	Mid brainedial portion	Histology, HE, PAS, coronal sections	[22]
Rat	Wistar	M	NS	28	I, RH^b^	i.p	1000 or 1500 tachyzoites	49 days	Mid brain	Histology, HE, PAS, coronal sections-10 rats	[22]
Mice	BALB/c	F	7 w	6	II, Prugniaud^a^	i.p.	400 tachyzoites		More cysts in amygdalar region	Histology-data pooled, bioluminescence	[21]
Mice	CD, Outbred	M	mo	5	NS, HIF^c^	oral	10 tissue cysts	18 weeks	No preference	Coronal sections, histology, HE, 1600 sections per mouse	[23]
Mice	C57BL/6J	F	7 w	19	II, ME49^d^	i.p.	100, 1000, 100,000	9 weeks^e^	No tropism	Coronal sections-40 μm, fluorescence microscopy	[24]
Mice	C57BL/69H-2^b^)	NS	10–12 w	6–14	II, ME49^d^	i.p.	20 tissue cysts	3 weeks, 6 weeks	At 3 weeks, tissue cysts more in hippocampus, same trend at 6 weeks but results not conclusive	tissue cysts counted in homogenate of brain	[25]
Mice	Swiss webster	F	NS	4	II, ME49^d^	i.p.	10 tissue cysts	6 months NS		Homogenate of brain, 20μl tested, 213 tissue cysts measured	[26]
Mice	STR, outbred	NS	2 mo	65	SRA	s.c.	12 tissue cysts	7 days-22 mo	Pathogenesis described. cyst rupture not observed^f^	HE, TEM, IHC	[27]

^a^ Strain isolated in 1964 from human fetus, genetically modified to express firefly luciferase and green fluorescent protein.

^b^ Strain isolated in 1937 from the brain of a child. It can no longer induce oocyst formation in cats and some lines of this parasite disappear in animals 21 days p.i.

^c^ Isolated in 1993 from the brain of a HIV-positive patient.

^d^ Isolated in 1950’s from diaphragm of sheep. Genetically modified to express green fluorescent protein.

^e^ 14.3%-mortality-10,000; 19.2%-mortality 100,000.

^f^ Of 32 mice studied 1–22 mo p.i., few contained glial nodules with immunoreactive debris/organisms but most nodules did not contain *T*. *gondii*. There was no evidence for new tissue cyst formation.

M = Male, F = Female, N = Number, NS = Not stated, w = Week, mo = Month, i.p. = Intra peritoneal, HE = Hematoxylin and Eosin. PAS = Periodic Acid-Schiff reagent.

**Table 2:** Summary of studies on size of tissue cysts in the brains of rodents inoculated with *T*. *gondii*.

Host				*T*. *gondii* strain	Tissue cyst data				References
Host	Strain	Sex	No.		P.I.	Methods	No. measured	Size	
Mice	BALB/c	M	5	^a^ME49	1, 2, 6 mo	Brains fixed, cryosectioned, FITC-DB	NS	1 mo-7-20 μm,2 mo-3-30 μm,6 mo-5-25 μm	[28]
Mice	STR, Outbred, albino	NS	18	^b^SRRA	3, 6, 12, 18, 22 mo	Brains fixed, 1 μm sections stained		3 mo-15-30 μm,6 mo-20-50 μm,12-22-30-50 μm	[29]
Mice		NS	20	^c^Burk	4, 8, 12, 16 and 24 weeks	Brain homogenate in saline	100 at each time point	8 weeks-38 μm,12 weeks-40 μm,16 weeks-41 μm,24 weeks-42 μm	[30]
Mice	CBA/J	F	10	^ME49^	3,4,5,6,8 weeks	Percoll isolated tissue cysts	630	Tissue cyst size relatively constant at 5 points studied	[31]
Rat	Sprague-Dawley	F	35	^d^VEG	75 days	Brain, formalin fixed, histological sections, HE stain	224	Up to 50 μm, most 22.5 to 30 μm	[32]

^a^ Type II, 200 tachyzoites, subcutaneously.

^b^ Beverley strain from rabbit, genotype unknown, 12 tissue cysts, subcutaneously.

^c^ Genotype unknown, tissue cysts, subcutaneously.

^d^ Type III, 1–1 million oocysts, orally.

M = Male, F = Female, NS = Not stated, mo = Month, P.I. = Post infection.

*Toxoplasma gondii* is a highly successful parasite. The definitive cat host is thought to principally become infected with *T*. *gondii* by carnivorism. Alterations observed in rodent behavior favor its success in transmission. *Toxoplasma gondii* infected rodents lose their fear of cats, and thus are attracted rather than repelled by feline urine, including wild felids, thus avoiding the feline territory marked by cats [21,33–36]. Some studies suggest that dopamine plays a role in this behavior although the mechanism has not been defined [37–39]. Tissue cyst location has been proposed to be a factor in observed behavior changes, for example, tropism for a specific brain region. The distribution of *T*. *gondii* in different brain regions is of clinical/biological significance because of its association with psychomotor disorders [40–42]. Studies in rodents pertaining to this topic are summarized in Table 1.

In the present study, we have reevaluated the rat model of toxoplasmosis, using rats of the same age, strain, and sex, after oral inoculation with oocysts (natural route and natural stage of infection) of 11 *T*. *gondii* strains of seven genotypes (Table 3). We euthanized rats at two months post inoculation (p.i.) and investigated the association of parasite genotype with tissue cyst distribution, location, size, and lesions in brain and eye. We were particularly interested in tissue cyst rupture and the fate of the released bradyzoites.

**Table 3:** Experimental toxoplasmosis in rats^a^ inoculated orally with oocysts of 11 strains of *T*. *gondii* of different genotypes.

*T*. *gondii* strain (Group no.)	ToxoDB PCR-RFLP genotype # (Conventional)	Origin Country	Host	References	No. of oocysts^e^	Died	Day killed	Rat nos.
GT1 (1, 2)	#10 (Type I)	USA	Goat	[43]	100000	8/10^b^	65	6157,6158
(52)					1000	0/5	62	6228–6232
TgCtCO2 (3, 4)	#28 (Atypical)	Colombia	Cat	[44]	100000	5/10	62	6159–6163
(53)					1000	0/5	66	6233–6237
ME49 (5, 6)	#1 (Type II)	USA	Sheep	[45]	10000	0/10	62	6164–6173
TgNmBr1 (7, 8)	#1 (Type II)	Brazil	Rabbit	[46]	100000	0/10	65	7174–6183
VEG (9, 10)	#2 (Type III)	USA	Human	[47,48]	100000	0/10	63	6184–6193
TgGoatUS4 (11, 12)	#2 (Type III)	USA	Goat	[49]	100000	6/10	63	6194–6197
(54)					10	0/5	62	6243–6247
TgBbUS1 (13, 14)	#147 (Atypical)	USA	Black bear	[50]	100000	0/5	66	6243–6247
(56)					1000	1/5 ^c^(day 10)	62	6248–6252
(55)					100	0/5^d^	62	6243–6252
TgPigUS15 (15, 16)	#8 (Atypical)	USA	Pig	[51,52]	10000			
TgRabbitBr1(17, 18)	#19 (Atypical)	Brazil	Rabbit	[46]	10000	10/10		6144 (day 7)
					100	0/5	62	6258–6263
					10	0/5	62	6253–6267
TgCtPRC3 (19, 20)	#18 (Atypical)	China	Cat	[53]		0/10	66	6208–6217
CT1 (59)	#10 (Type I)	USA	Cattle	[54]	10000			D6268-6272
(60)					1000			D6263-6267

^a^ All rats were 46 day old on day of *T*. *gondii* inoculation.

^b^ Medicated with sulfadiazine I drinking water day 7–10 p.i.

^c^ Medicated with sulfadiazine I drinking water day 10–11 p.i.

^d^ Medicated with sulfadiazine I drinking water day 15–19 p.i.

^e^ Mouse infective units, not actual count.

## Materials and Methods

### Ethics statement

All procedures were approved by the Beltsville Area Animal Care and Use Committee, United States Department of Agriculture.

### Rats

Sprague Dawley (*n* = 150) female rats (SAS-SD, Code) were used in two trials. They were obtained from Charles River, New York. In the first trial, 100 rats were inoculated orally with sporulated oocysts, with 10 rats as uninoculated controls. The rats were observed twice daily for illness. Unexpectedly, 39 rats died overnight of acute toxoplasmosis, without obvious clinical signs (Table 3). The rats were given anti-*T*. *gondii* therapy (see Table 3) when clinical signs were anticipated or cagemates had died. Therefore, a second batch of rats was inoculated with lower doses, 38 days later (Table 3). In both trials, rats were inoculated when they were 42 days old, and they were from the same source. Rats were housed in groups of five in rat cages and were given water and rodent pellets ad lib.

### *Toxoplasma gondii* strains

Eleven strains were used in this investigation. Oocysts were obtained by feeding infected tissues to *T*. *gondii*-free cats as described [1,55]. Oocysts were sporulated and stored in 2% sulfuric acid at 4°C. For inoculation into rats, sulfuric acid was removed by centrifugation and oocysts were inoculated orally by a stomach tube. The number of infective oocysts was determined by bioassay in mice [1]. For this, 0.5 ml of the inoculum fed to rats was diluted serially 10-fold. A 10^−4^ dilution of inoculum of each strain was infective to five out of five mice. Thus, there were at least 10,000 infective oocysts in each inocula. The doses in Table 3 refer to mouse infective units and not the visual count because the oocysts had been stored for various times before feeding rats. Oocysts for this experiment had been collected at various times and stored in refrigerator. The titrations in mice were made after the experiment when the rats had died unexpectedly. We did not have an estimate of viable oocysts in the inocula before feeding them to rats. In hindsight it would have been ideal to titrate oocysts before infection of rats. Bioassay in mice sometimes takes 3 months. With respect to influence of chemotherapy and the fate of tissue cysts, only a few rats had received chemotherapy, and it is for this reason the individual rats are indentified in Table 3. As such, the drugs used have no known effect on tissue cysts, they are active only against tachyzoites.

### Necropsy examination

The experiment was terminated two months post inoculation (p.i.) as planned, at which point all rats were clinically normal. All rats, including controls, were euthanized between 62–66 days after start of the experiment using the CO_2_ gas anesthesia followed by thoracotomy. After obtaining blood from the chest cavity, samples of tongue and skeletal muscle (from thigh) and both eyes were fixed in 10% buffered formalin. Entire brain from olfactory bulb to cervical spinal cord of each rat was removed. The brain was sliced longitudinally, at the midline. The entire one-half of the brain was saved for histology. From the second half, approximately 2–3 mm longitudinal slice was also saved for histology and the remainder of brain was not fixed (S1 Fig).

### Tissue cyst enumeration

Unfixed brain (S1 Fig) was homogenized in 1.5 ml saline (0.85% NaCl) with a mortar and pestle and passage through a 22 gauge needle and syringe; to this homogenate an equal part of 10% formalin was added and stored at room temperature for tissue cyst size measurement. For tissue cyst size, 10 μl of brain homogenate was dispensed onto a glass slide and covered with a 22 x 22 mm No.1 glass coverslip. Using this volume under a coverslip, tissue cysts were floating and not compressed. Measurements were made with micrometer in eye piece at 400x magnification.

### Histological examination

For histology, tissues were fixed in 10% buffered formalin. Tissues from each rat were processed for embedding in three paraffin blocks, a block of muscle (a cross section of tongue and myocardium at mid point, and 2 x 1 cm pieces of skeletal muscle from rear limb), one block containing both eyes, and the third block of brain containing two sagittal sections of both halves of the brain. Sections of brains were stained with hematoxylin and eosin (HE) or periodic acid-Schiff reagent (PAS) counter stained with hematoxylin (PASH) (S1 Fig). Sections of muscles were stained with PASH.

Histological sections were made from the middles of eyes, at the point of entry of optic nerve. Both eyes were embedded in one block and four to six sections were made from each block. Eyes of 16 rats (all 10 infected with ME49 strain, and 6 infected with TgNmBr1) had been discarded inadvertently before histological examination.

### Immunohistochemical staining (IHC)

Histological sections were reacted with rabbit polyclonal *T*. *gondii* [1], and with anti-bradyzoite (BAG1) antibodies and counter staining with hematoxylin as described previously [56]. The staining procedure described by [1] was followed for all sections.

### Tissue cyst distribution in histological sections of brain

Both sagittal sections for 108 infected rats stained with BAG1, and PASH were scanned for data analysis at the Virtual Pathology Centre at the University of Leeds (http://slides.virtualpathology.leeds.ac.uk) (S2 Fig). The brain scans were divided into regions cerebellum, rhombencephalon, mesencephalon, hippocampus, thalamus, hypothalamus, subpallium, frontal cortex, olfactory bulb, and the colliculus (inferior and superior), based on the rat brain atlas [57] (S1 Table). Individual tissue cysts were then located visually and counted manually on the scanned slides. The total number of tissue cysts for each region (for each rat) was tallied. To assess tissue cyst density, the brain regions were measured for 20 representative brains chosen as every fifth animal of the study and the relative area of each region determined using ImageJ analysis (S2 Table). The size of the brain region was reported as a percentage of the whole brain slice and these percentages were used to calculate the tissue cyst density.

### Statistical analysis

Data was analyzed using IBM SPSS statistics software (version 22). Normality of the dataset was tested using Shapiro-Wilk test and the distribution was found non-normal. Therefore, non-parametric tests were used for analysis with the Kruskal-Wallis and Mann-Whitney tests.

### Degree of encephalitis

An attempt was made to record inflammatory lesions in the brain as +, ++, and +++. Three plus lesions were present throughout the brain. Category 2 (++) lesions were multiple foci, whereas category 1 (+) lesions were solitary.

### Antibodies to *T*. *gondii*

Antibodies to *T*. *gondii* in rat sera were determined using the modified agglutination test (MAT) as described [58].

## Results

### Clinical

In the first trial, 39 of 50 rats fed oocysts of five *T*. *gondii* strains (8 of 10 GT1, all 10 TgBbUS1, 5 of 10 TgCtCO2, all 10 TgRabbitBr1, and 6 of 10 TgGoatUS4) died of acute toxoplasmosis (enteritis and pneumonia) over the weekend between six and eight days p.i. (Table 3). The two surviving rats fed GT1 strain were given sulfadiazine sodium in drinking water (1mg/ml) for two days, day seven and eight p.i. In the second trial, one of five rats fed oocysts of the TgBbUS1 strain died of toxoplasmosis on day ten p.i. (Table 3). All tissues of two rats (1 TgBbUS1, Rat D6143 and 1 TgRabbitBr1, Rat D6144) that died of acute toxoplasmosis were processed for histological examination. Rats fed oocysts of the TgBbUS1 were medicated with sulfadiazine, 10–19 days p.i. (Table 3).

Rats that survived the first two weeks after feeding oocysts appeared healthy by clinical observation.

### Acute toxoplasmosis

**Fig 1:**
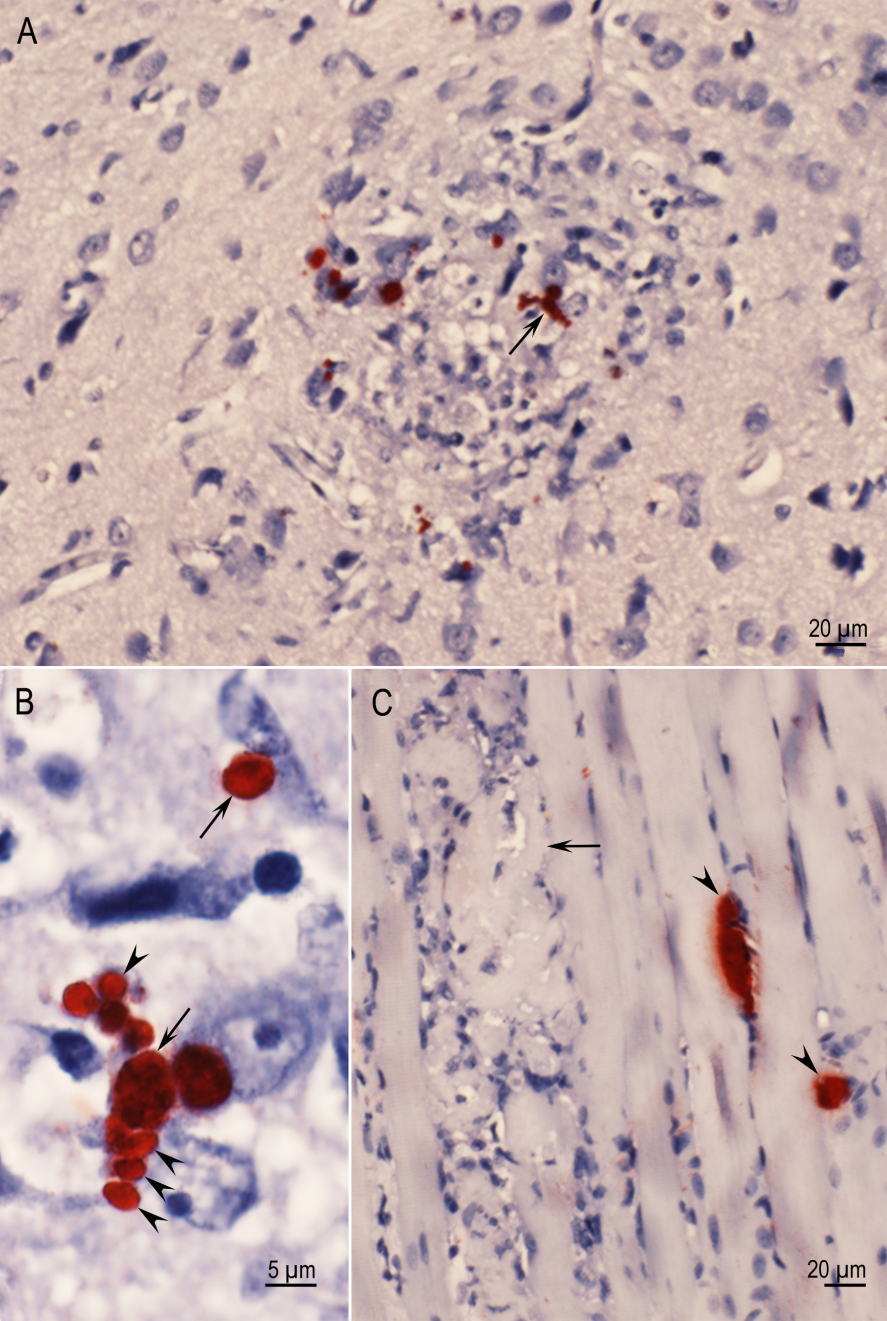
Acute toxoplasmosis and tissue cyst formation in cerebrum of Rat D6143 infected with the TgBbUS1 strain, 7 days p.i. IHC staining with BAG1 T. gondii antibodies. (A) A focal area of necrosis, the genesis of a glial nodule, individual bradyzoites and small tissue cysts (arrow). Several tachyzoites are present in the lesion but are unstained. (B) Higher magnification of an area indicated by arrow in Fig 1A. Note individual bradyzoites (arrowheads) and small tissue cysts (arrows). (C) Necrosis (arrow) and two tissue cysts (arrowheads) in skeletal muscle.

**Fig 2:**
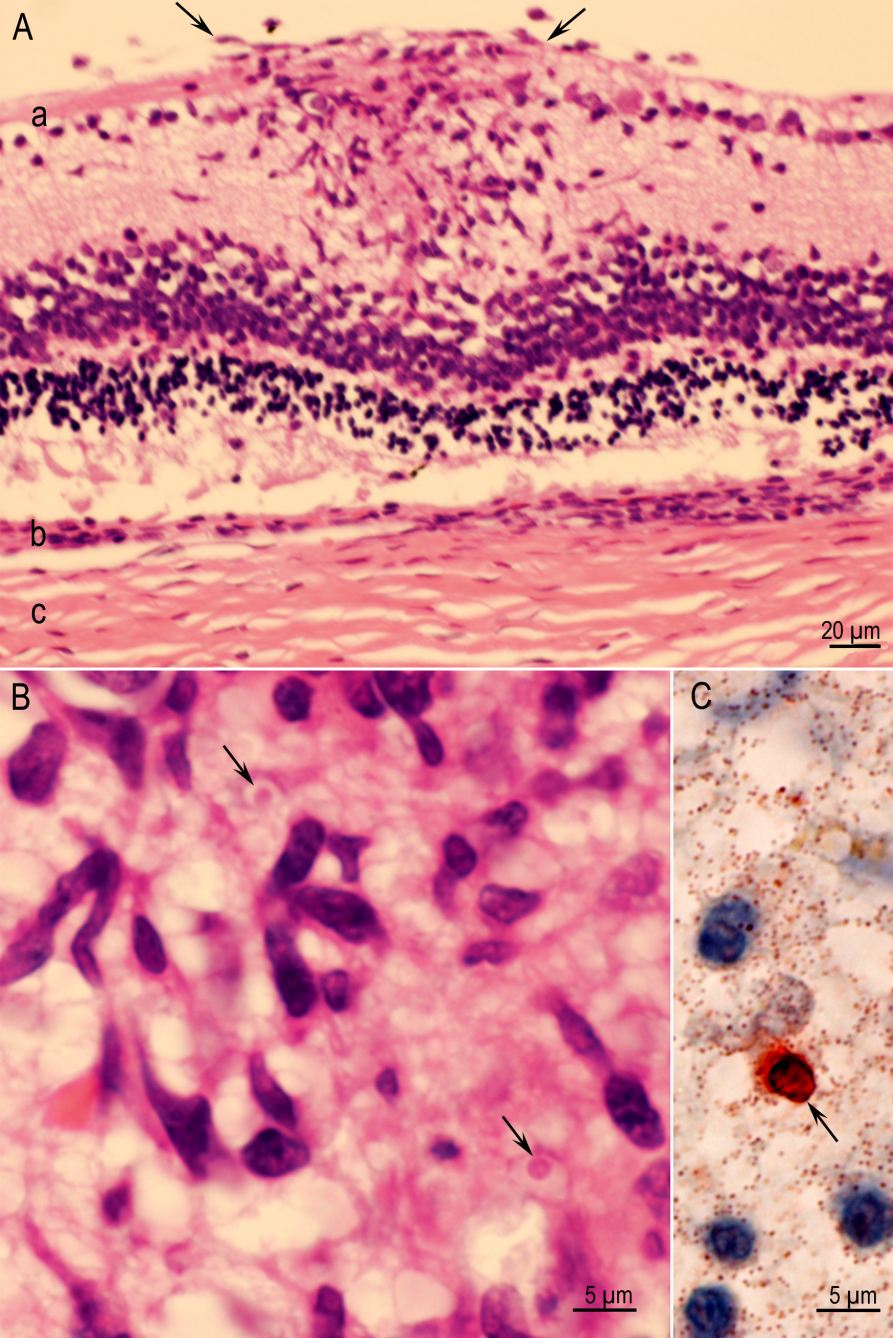
Focal inflammation in retina (a) of Rat D6143 infected with the TgBbUS1 strain, 7 days p.i. (A) Necrosis, and inflammatory focus, primarily affecting the inner retinal layers, bulges into the vitreous chamber (arrows). The choroid (b) and sclera (c) are not affected. HE stain. (B) Higher magnification of Fig 2A with two tachyzoites (arrows). (C) A tachyzoite (arrow) in the inflammatory focus. IHC stain with polyclonal *T*. *gondii* antibodies. This tachyzoite is larger in size than the tachyzoites stained with HE in Fig 2B because tachyzoites swell during immunostaining.

Rats that died between seven to ten days p.i. had enteritis, pneumonia, encephalitis, and myocarditis with tachyzoites in lesions. Individual bradyzoites and tissue cysts were seen in the brains and skeletal muscles of both rats sectioned (Fig 1). A focus of retinitis with tachyzoites was seen in one eye (Fig 2).

### *Toxoplasma gondii* serology results

Antibodies to *T*. *gondii* were found in sera of all inoculated rats (1:500 dilutions) that were euthanized two months post inoculation.

### Tissue cyst density versus distribution

**Fig 3:**
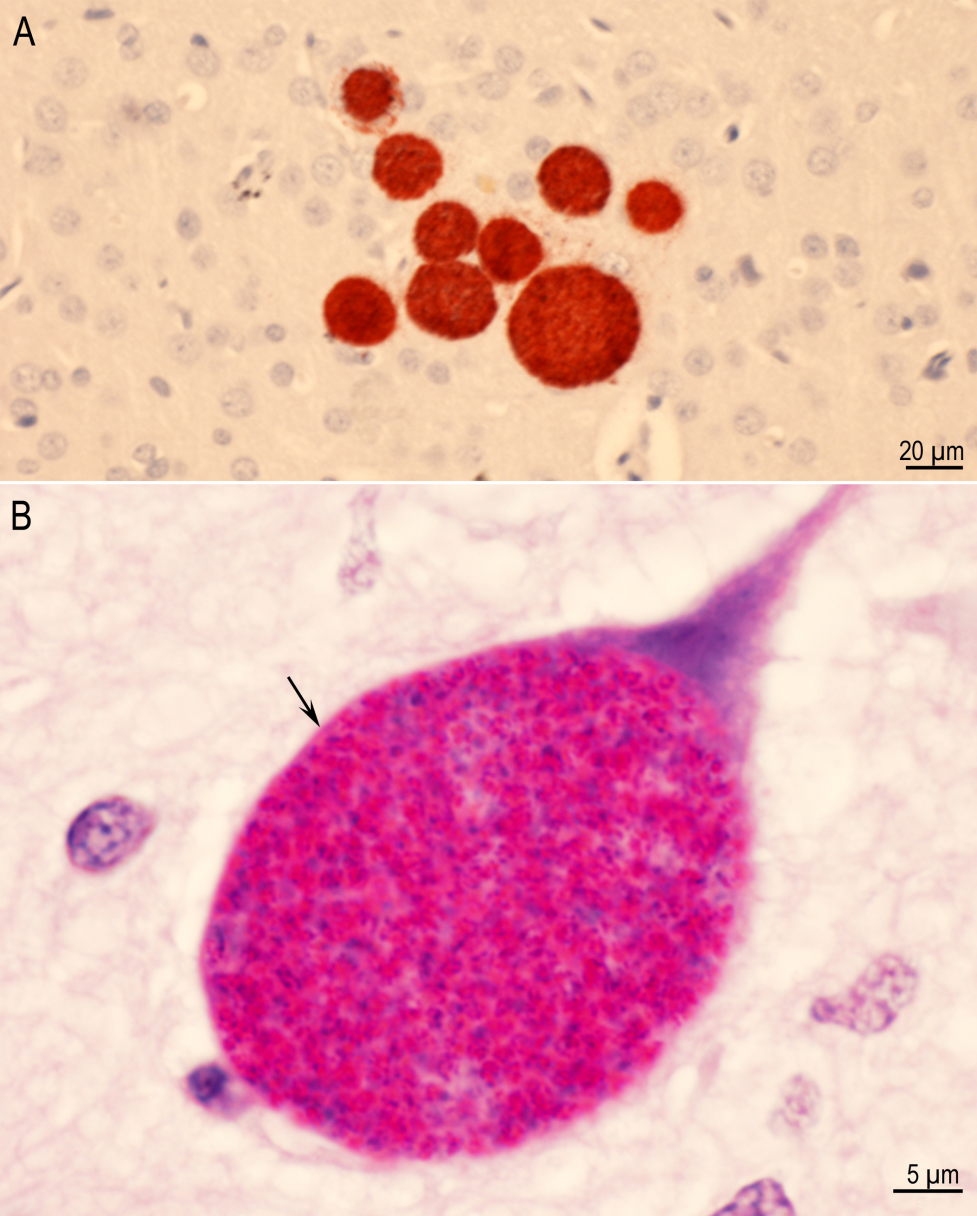
Tissue cysts in sections of brains of rats. (A) A group of nine tissue cysts without host reaction. IHC staining with BAG1 *T*. *gondii* antibodies in Rat D6255 infected with the TgRabbitBr1 strain (B) Intraneuronal tissue cyst with thin PAS-negative cyst wall (arrow) enclosing numerous bradyzoites that are stained red. Rat D6249, Strain TgBbUS1. PASH stain.

Tissue cysts were found in all regions of the brain in chronically infected rats (Fig 3).

**Fig 4:**
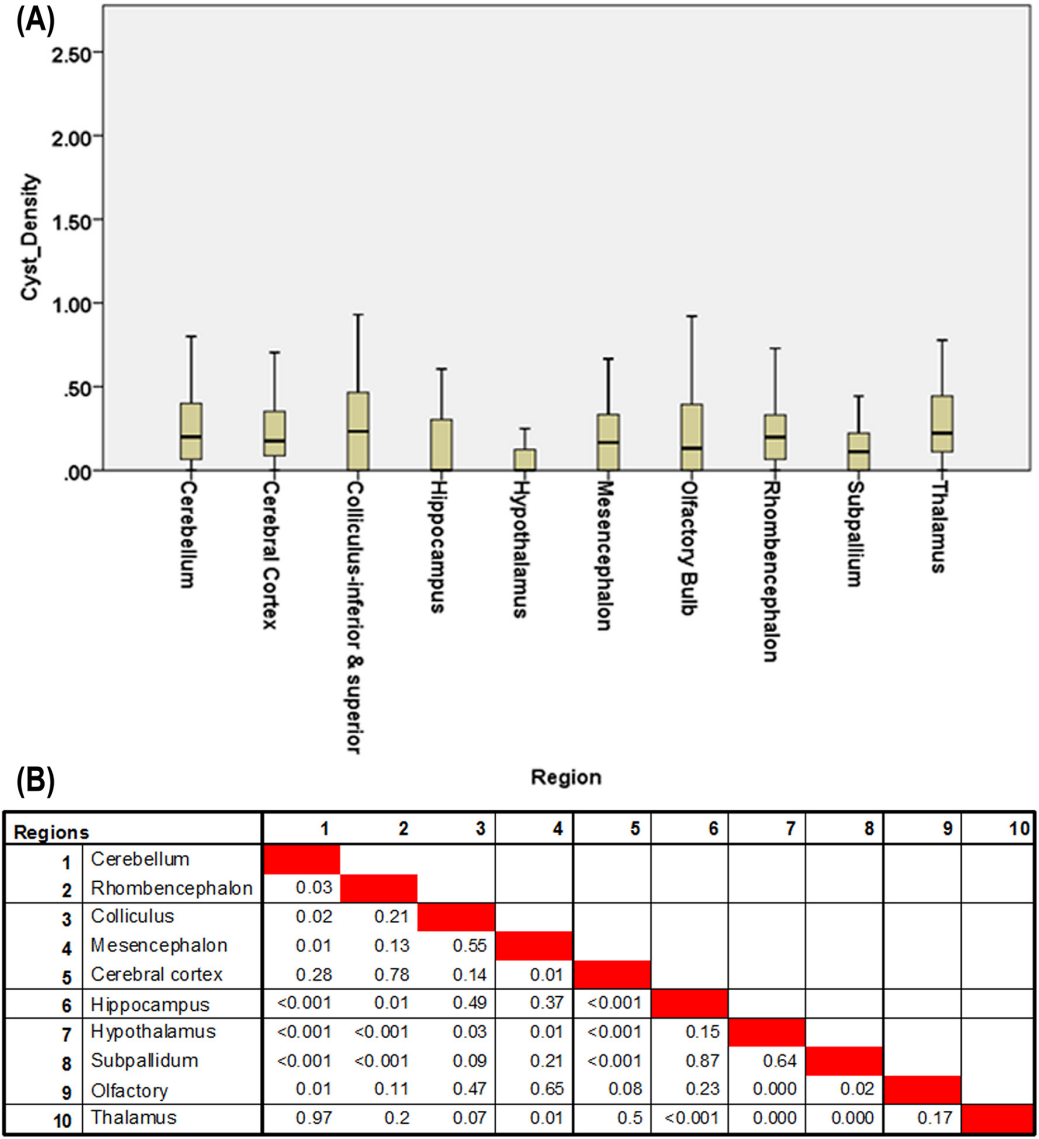
Tissue cyst distribution and pair wise comparison of the brain regions for infected rats. (A) Box and whisker plot of tissue cyst densities for the regions in the mid-sagittal sections from the 108 rats (top). Table of statistics for comparison between pairs of regions using Mann-Whitney (bottom). SPSS Graph Builder (version 22) was used to draw the boxplot. (B) P-values for comparisons of tissue cyst densities in regions using the Mann-Whitney test. The Bonferroni corrected p-value cutoff for multiple testing is 0.0011 (0.05/45).

The tissue cyst density in different brain regions varied extensively between the 108 rats examined with many regions highly infected in some animals. Overall, the colliculus was the most highly infected although there was a large amount of variability with three rats (Fig 4) considerably higher than the others. Based on comparison between regions (pairs compared using Mann-Whitney non-parametric test), 12 of the regional comparisons were significant, taking into account Bonferroni multiple testing corrections. The cerebral cortex, thalamus, and cerebellum had higher tissue cyst densities and the hypothalamus, subpallium, and hippocampus had lower tissue cyst densities. The rhombencephalon had a higher density than the hypothalamus or subpallium. The hypothalamus was lower than most of the regions particularly the cerebral cortex, thalamus, cerebellum, rhombencephalon, and olfactory.

Interestingly, two of the 11 strains analyzed, TgRabbitBr1 and TgCTPr-C3 showed a significant difference between brain regions (p < .003, Kruskal Wallis test) and strain CT-1 was borderline significant (p = 0.05, Kruskal Wallis test). Strains TgRabbitBr1 and TgCTPr-C3 had higher tissue cyst densities in the colliculus and olfactory bulb and lower density in the hippocampus. Strain CT-1 also had an elevated tissue cyst density in the olfactory bulb and reduced density in the hippocampus but, in contrast to the other two strains, had low tissue cyst density in the colliculus. There was not a significant difference between regions in the other strains analyzed including the two common lab strains ME-49 and VEG. This may have been due to the restricted number of animals examined (n = 9 per strain) or weak tropism for these strains.

### Tissue cyst density and size by *T*. *gondii* strain

**Fig 5:**
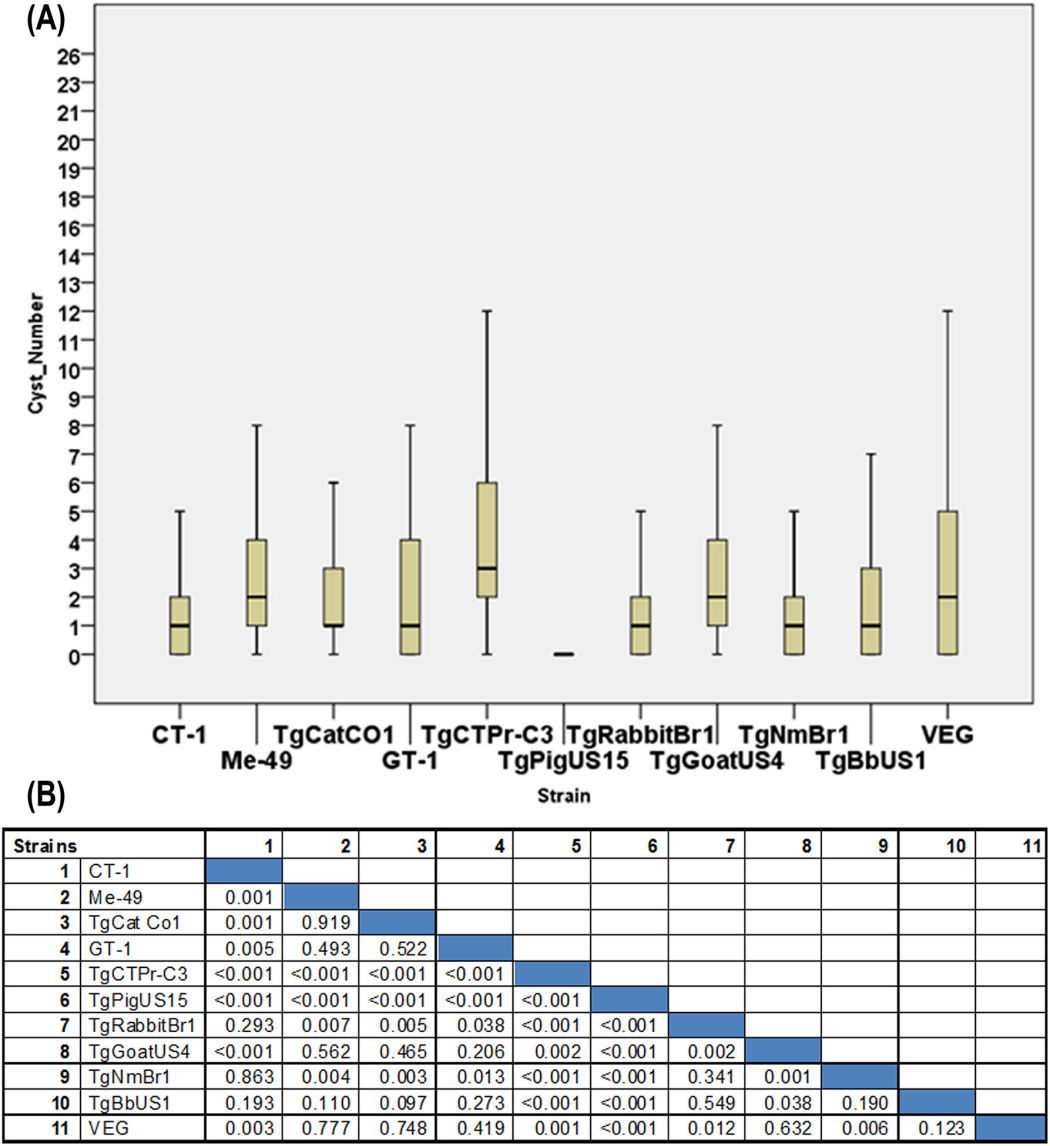
Comparison of tissue cyst counts between strains of *T*. *gondii*. (A) Box and whisker plot of tissue cyst number from the mid-sagittal sections for the 108 rats with eleven strains (n = 9–10 rats per strain). SPSS Graph Builder (Version 22) was used to create the boxplot. (B) Table of statistics for comparison between strains using Mann-Whitney test as Fig 4. Bonferroni multiple testing correction p-value cutoff is (0.05/55).

The highest density of tissue cysts was observed in brains of rats infected with the TgCTPr-C3 strain (Fig 5, Table 4). The tissue cyst count for the TgCTPr-C3 strain was higher than any of the other strains tested although this trend was not statistically significant for the TgGoatUS4 and VEG strains. Only a few tissue cysts were found in rats inoculated with the TgPigUS15 strain, and data were consistent both in histological sections and in homogenized brain (Table 4).

**Table 4:** Measurements of tissue cysts from rat brains infected with different strains of *T*. *gondii*.

*T*. *gondii* strain	Genetic type (ToxoDB PCR-RFLP genotype #)	No. of tissue cysts measured	Size (diameter) in μm	
			Mean	SD
TgGoatUS4	Type III (#2)	550	36.14	4.22
GT1	Type I (#10)	688	38.15	2.95
TgNmBr1	Type II (#1)	373	40.78	3.06
CT1	Type I (#10)	204	40.92	8.83
VEG	Type III (#2)	900	42.48	2.24
TgCTPrC3	Atypical (#18)	625	43.54	1.57
TgBbUS1	Atypical (#147)	625	49.19	3.41
TgRabbitBr1	Atypical (#19)	177	49.23	8.38
ME49	Type II (#1)	632	49.65	2.54
TgCatCo1	Type I (#28)	476	51.39	4.91
TgPigUS15	Atypical (#8)	12	65.33	11.34

Tissue cysts were 36 to 75 μm in size in homogenates of brains (Table 4). There was no apparent correlation of tissue cyst size and genotype.

### Tissue cysts in muscles

Tissue cysts were not found in the histological sections of heart and skeletal muscle of any rat (Table 5). Tissue cysts were seen in sections of tongue of 20 rats (Table 5).

**Table 5:** Summary of lesions in extra ocular tissues of rats inoculated orally with 11 strains of *T*. *gondii*^a^.

*T*. *gondii* strain	GT1	TgCatCo1	ME49	TgNmBr1	VEG	TgGoatUS4	TgBbUS1	TgPigUs15	TgRabbitBr1	TgCtPRC3	CT1
**Brain** Lesions											
Score	1.2	1.8	1.5	0.9	1.9	1.5	1.7	0.6	1.3	2.2	0.6
Glial nodules with necrosis	2		3		5	4	5	1	2	6	
Cyst rupture	1	3	3	2	3	4	2	0	2	5	0
**Heart**											
Lesion	1	5	0	1	1	4	3	0	0	0	0
Cyst	0	0	0	0	0	0	0	0	0	0	0
**Tongue**											
Lesion	3	3	0	1	2	3	2	0	2	1	0
Cyst	4	1	4	1	3	3	0	0	2	2	0
**Skeletal muscle**	0	0	0	0	0	0	0	0	0	0	0

^a^ No of rats with lesions out of 10 rats per *T*. *gondii* strain.

### Lesions in brain

**Fig 6:**
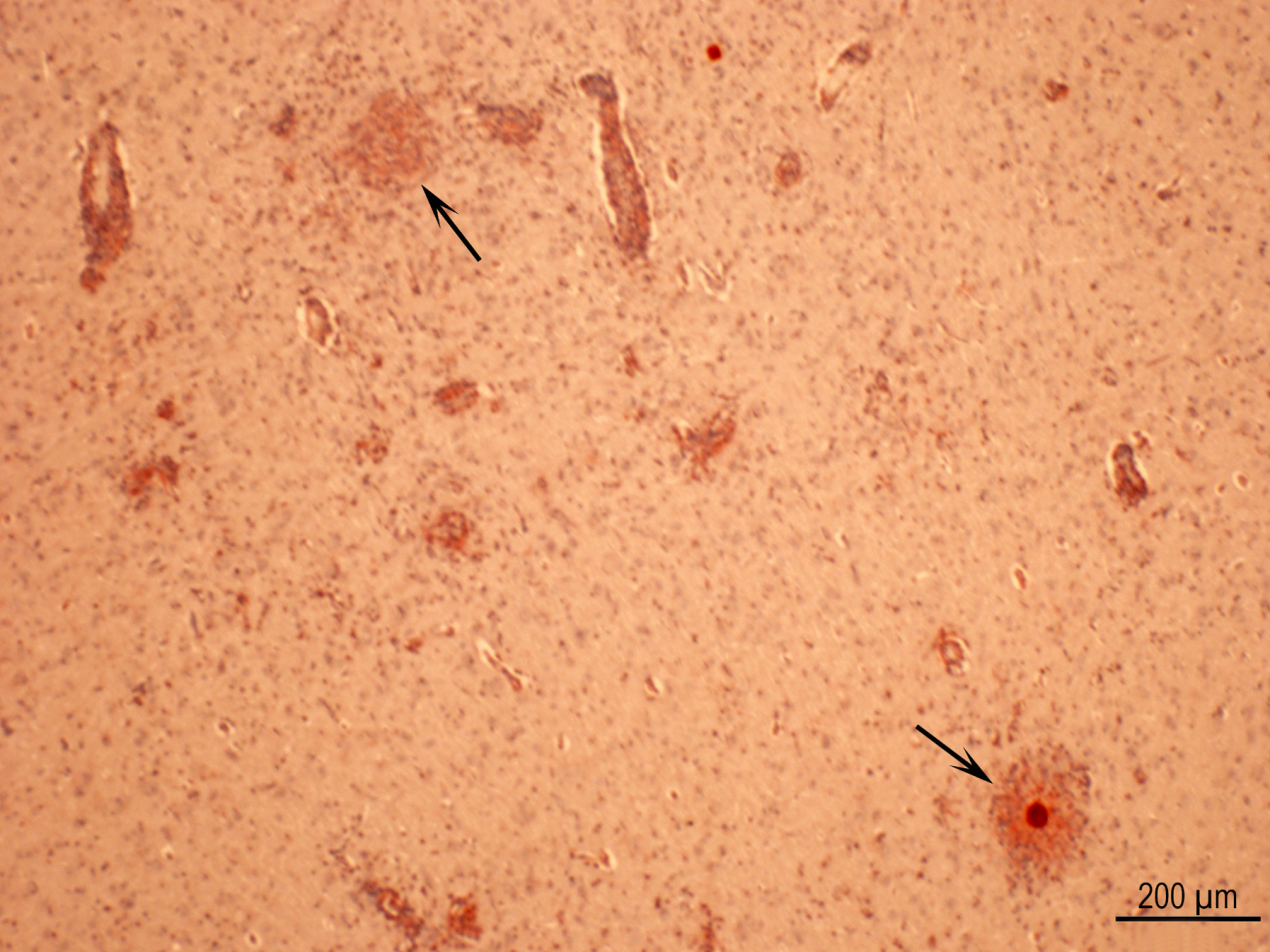
Multiple necrotizing foci in cerebrum of Rat D6162 infected with the GT1 strain. Two foci have degenerative tissue cysts (arrows). IHC staining with polyclonal *T*. *gondii* antibodies.

**Fig 7:**
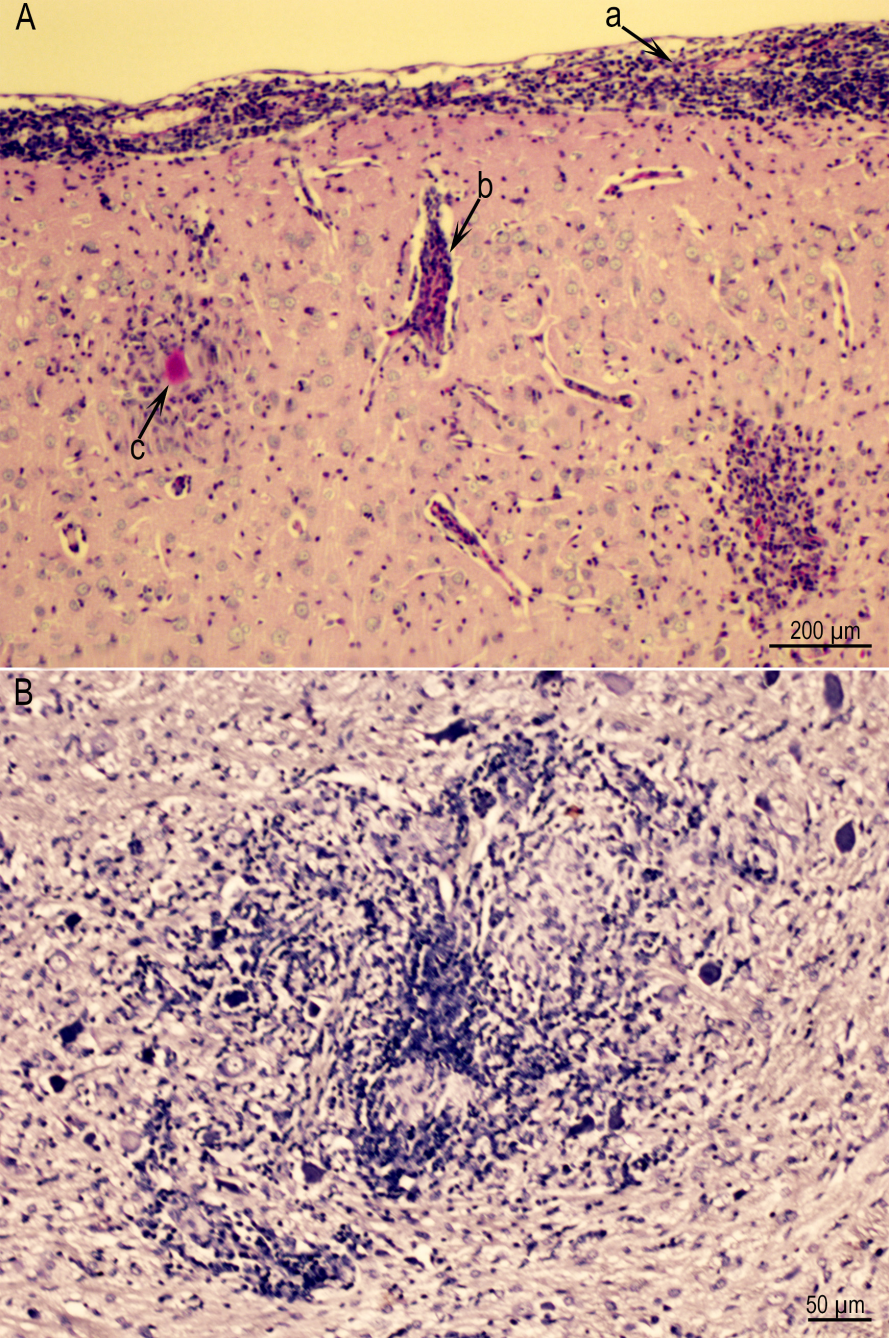
(A) Meningoencephalitis in Rat D6157 infected with the TgCatCo3 strain. Note meningitis (a), perivascular infiltration of leukocytes (b), and a glial nodule with a degenerative tissue cyst (c). PASH stained. (B) A large inflammatory focus, probably resulting from the host reaction to degenerative tissue cysts in Rat D6162 infected with theGT1 strain and HE stained.

**Fig 8:**
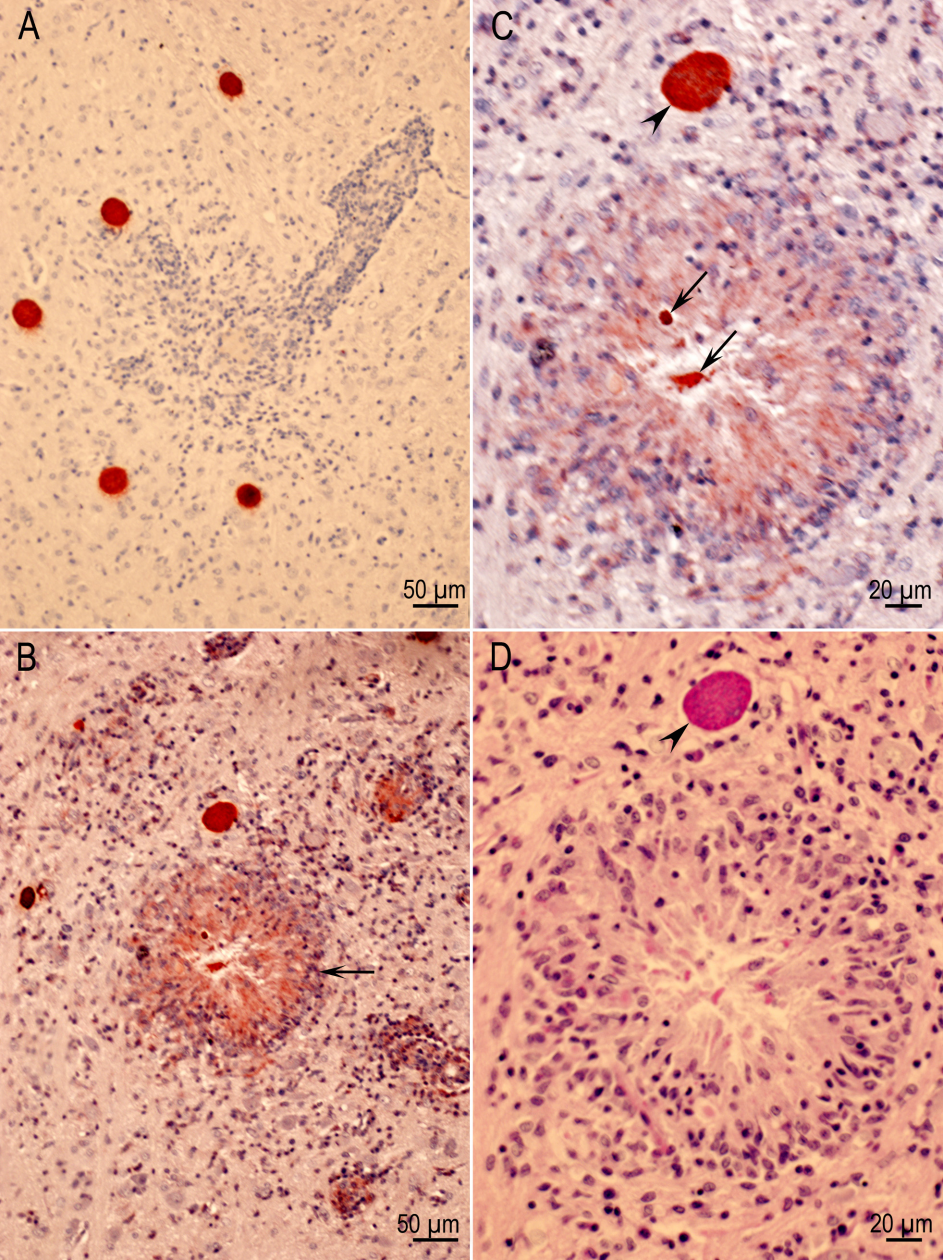
Examples of encephalitis with intact and degenerative tissue cysts. For Rat D6162 infected with the GT1 strain: (A) Vasculitis with five intact tissue cysts at the periphery of the lesion. IHC staining with *T*. *gondii* BAG1 antibodies and hematoxylin counterstain. (B) Several glial nodules, one with a degenerative tissue cyst (arrow). (C) Higher magnification of focus arrowed in Fig 8B. Note immunoreactive debris (arrows) in focus of necrosis focus. One intact tissue cyst (arrowhead) is at the periphery of the lesion. (D) The same lesion as in Fig 8B. An intact tissue cyst (arrowhead) is present at the periphery of glial nodule with central necrosis. There are no visible bradyzoites. PASH stain.

**Fig 9:**
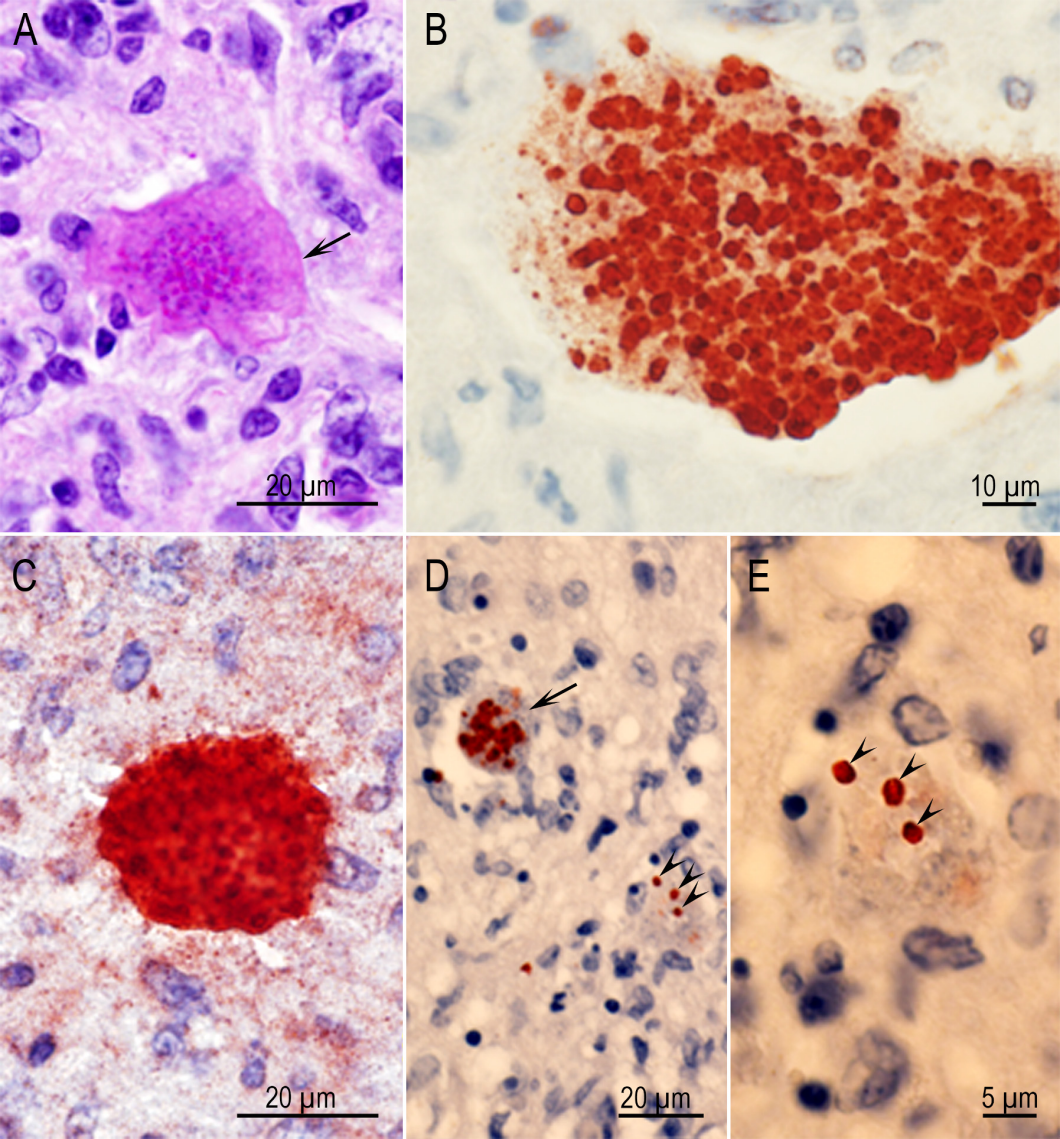
Examples of degenerative tissue cysts in brains of rats. (A) Higher magnification of the lesion in Fig 7D with Rat D6157 infected with strain TgCatCo3. The cyst wall (arrow) is visible but bradyzoites have degenerated. (B) Bradyzoites in different stages of degeneration in Rat D6163 infected with the TgCatCo3 strain. The tissue cyst wall is not visible. IHC staining with BAG1 *T*. *gondii* antibodies. (C) Intensely stained bradyzoites in Rat D6162 infected with the GT1 strain. IHC staining with polyclonal *T*. *gondii* antibodies. (D, E) Two tissue cysts in different stages of degeneration in Rat D6162 infected with the GT1 strain. The outline of the tissue cysts is visible in one (arrow) and not in the other (arrowheads). The bradyzoites (arrowheads) appear to be intact.

Inflammatory lesions were present in the brain of most infected rats. The most severe lesions consisted of meningitis, perivasculitis, glial nodules with or without central necrosis, degenerating tissue cysts, and individual organisms (Figs 6, 7, 8 and 9). Average lesion scores are shown in Table 5.

Ruptured tissue cysts were found in 25 of 110 rat brains (Table 5). Tachyzoites were not demonstrable in any rat euthanized two months post infection.

### Lesions in eyes

**Fig 10:**
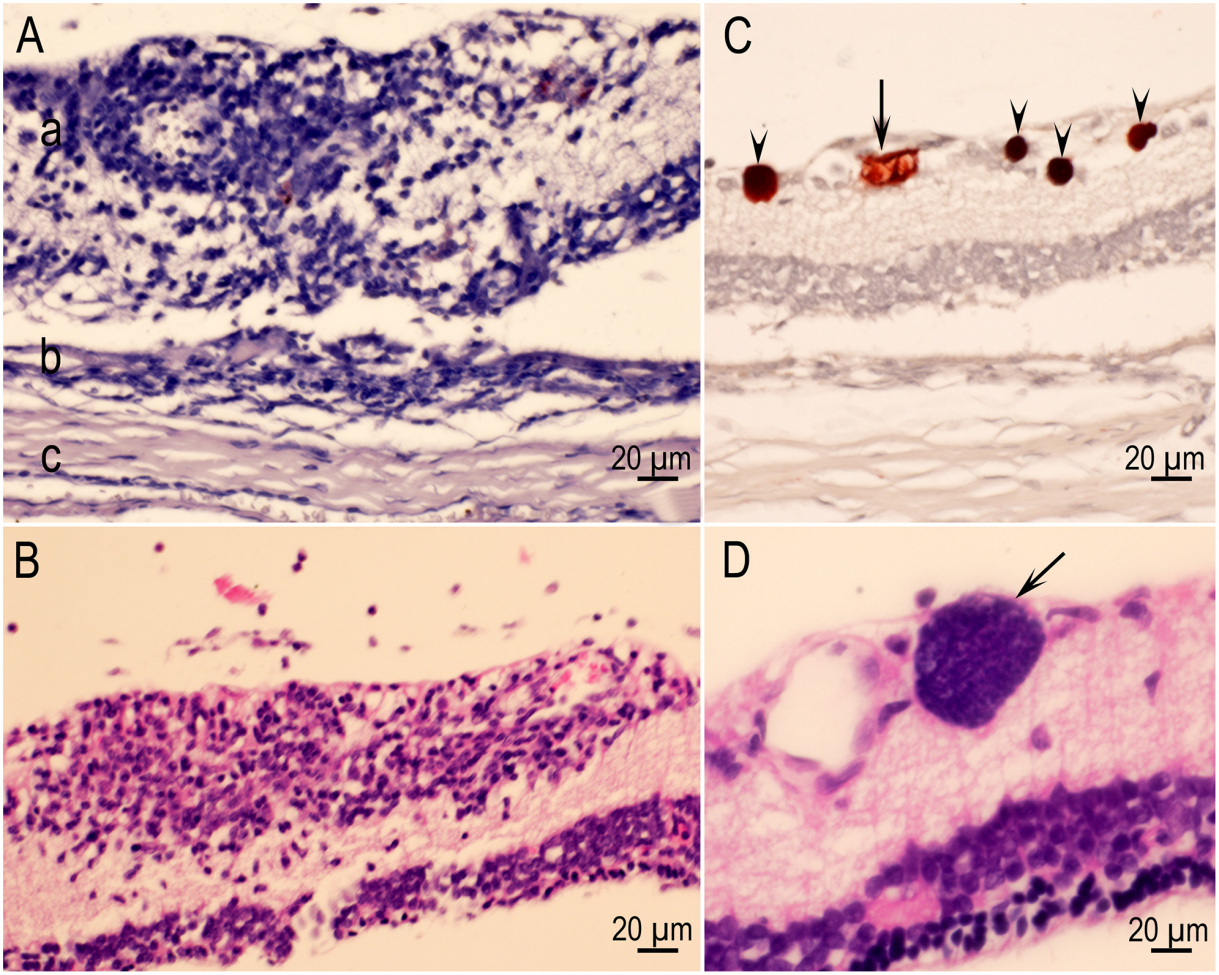
Retinitis in rats. (A) Inflammatory focus involving the entire width of the retina (a) and encroaching the choroid (b). The sclera (c) is unaffected. Rat D6185 infected with the VEG strain and HE stained. (B) A focally extensive area of inflammation (retinitis) composed of lymphocytes and few macrophages affecting primarily the inner layers of the retina. Rat D6243 infected with the TgBbUS1 strain and HE stained. (C) Four intact tissue cysts (arrows) and a probable degenerating tissue cyst (arrowhead) in retina. Rat D6186 infected with the VEG strain. (D) Tissue cyst (arrow) in retina without host reaction. Rat D6249 infected with the TgBbUS1 strain and HE stained.

Lesions were found in 23 of 92 eyes of rats euthanized at two months p.i. (Table 6) and in animals infected with all strains except the TgNmBr1 (in which only four rats were infected). In two rats both eyes were affected. The predominant lesion was focal inflammation in the retina (Fig 10). Tissue cysts were seen in the sclera of one and in the optic nerve of two rats. The choroid was not affected. Only tissue cysts, not active tachyzoite infections, were detected in rats examined two months p.i.

**Table 6:** Ocular lesions in rats orally inoculated with *T*. *gondii* oocysts.

*T*. *gondii* strain	No. of rats with ocular lesions/No. of rats	Retina				
		One eye	Both eyes	Tissue cysts alone	Tissue cysts and lesions	Lesions alone
TgBbUs1	6/10	4	2	0	5	1
VEG	4/10^a^	4	0	1	3	0
TgRabbitBr1	3/10^b^	3	0	1	2	0
TgGoatUS4	2/10	2	0	1	1	0
TgCtPrC3	2/10	2	0	2	0	0
CT1	2/10	2	0	0	1	1
GT1	1/8	1	0	0	1	0
TgCatCo1	2/10	1	0	0	1	1
TgPigUS15	1/10	1	0	1	0	0
TgNmBr1	0/4					

^a^ Tissue cyst in sclera of one and optic nerve of one rat.

^b^ Tissue cyst in optic nerve of one rat.

## Discussion

### Tissue cyst tropism

Because of the interest in behavioral effects of hosts infected by *T*. *gondii*, several authors have studied tissue tropism in the brains of chronically infected rodents, particularly the amygdalar region (Table 1, [59]). Two reports found tropism to the amygdaloid [21] or mid brain regions [22]. In the most extensive study of localization of tissue cysts [23], the entire brains of five mice at 18 weeks p.i. were sectioned 8 μm apart, and sections were examined microscopically after HE staining. Tissue cysts in 54 regions of brains from olfactory bulbs to hind brain were counted. The authors concluded that tissue cysts were distributed unevenly in all regions of the brain with no specific tropism to amygdaloid regions. They provided the first quantitative data with respect to number of tissue cysts per millimeter of each region of the brain [23]. Another report indicated that tissue cyst distribution was not random but did not find tropism for any specific region [24]. Yet, two other studies reported that tropism could vary with duration of infection. Tropism to the hippocampus region was more evident at three weeks p.i. versus at six weeks p.i. in one report [25] and to the amygdala region at two months compared with six months [28]. Whether these differences are related to techniques, host, parasite strain, route of infection, duration of infection, or number of rodents (Table 2) is not clear. For example, data were based on only a few (6 or less) animals in five of eight studies listed in Table 2. Additionally, data are based on four *T*. *gondii* (RH, ME49, HIF, Prugniaud) mouse-adapted strains, passaged for many years.

In the present study in rats there was no predilection for tissue cysts in the amygdala and although there were higher tissue cyst densities in some regions (i.e. colliculus) there was also significant variability between rats. This is similar to the observations in infected mice [23]. Method of infection and stage of parasite may also influence tropism.

### Size of tissue cysts in brain

The tissue cyst size is dependent on the duration of infection, the type of host cell parasitized, strain of the parasite, and the cytological method used for measurement. Little has been published with respect to the effects of host, parasite genotype, and *T*. *gondii* strain on the tissue cyst size [60]. Most of the published information is derived from infection in mice with mouse-adapted *T*. *gondii* strains (Table 2). Beverley (1958) [61] first provided data on the tissue cyst size. He measured volume of tissue cysts in saline homogenates of brains of mice inoculated subcutaneously with tissue cysts of the rabbit a (Beverley) strain. Tissue cysts grew uniformly up to 10 weeks, after which there was considerable variability in tissue cyst size, perhaps due to a second generation of tissue cysts [39]. Most tissue cysts were less than 40 μm in diameter and rarely greater than 58 μm. This paper was presented in a conference and details are missing and thus not included in Table 2. Van der Waaij (1959) [30] studied growth of tissue cysts in mouse brain homogenates in which 100 to 500 tissue cysts freed from brain tissue were measured at 4, 8, 12, 16, and 24 weeks p.i.; tissue cysts grew uniformly in size up to 12 weeks p.i., and then the growth remained relatively constant. Tissue cysts were up to 70 μm in diameter, but the mean diameter of 100 tissue cysts at 16 weeks p.i. was 42 μm. Ferguson and Hutchison (1987) [29] measured 140 tissue cysts with a minimum of 10 tissue cysts at 11, 21, and 28 days, and 3, 6, 12, 18, and 22 months p.i. in thin sections by electron microscopy. Tissue cysts were up to 20 μm in diameter at 28 days, up to 30 μm at 3 months, and up to 50 μm at six months. In addition to data in Table 2, Sullivan et al. (2013) [26] provided micrometery data on 213 tissue cysts in the brains of four mice, six months after intraperitoneal inoculation with 10 tissue cysts of the ME49 strain of *T*. *gondii*. They measured tissue cysts in two directions to account for the variability of the shape of tissue cysts (elliptical versus circular) and proposed an equation for measuring the volume. They concluded that the volume of tissue cysts was the same irrespective of the shape and the size was dependent on the number of bradyzoites. At six months p.i., tissue cysts in brain homogenate varied from 10–80 μm but most were less than 60 μm.

Dubey (1996) [32] measured tissue cysts in formalin-fixed histological sections of the brains of mice and rats between 72 to75 days after feeding the rats oocysts. Although the tissue cysts were up to 50 μm in diameter, most of them were approximately 30 μm in diameter. Tissue cysts in brains of rats were of the same size as in mice; infections in both hosts were given the same inocula.

The measurement of tissue cysts in formalin-fixed, paraffin-embedded sections provides a standard means of reporting results. The sizes of the tissue cysts vary a great deal when unstained tissue cysts are examined between a glass slide and coverslip, depending on the homogeneity of the brain suspension, the amount of fluid, and the pressure applied. In the present study, tissue cysts were measured in formalin-fixed brain homogenate so that enough material could be examined over time when dealing with samples from more than 100 rat brains. In the present study, tissue cysts from infected rats varied from 34–61 μm. The size did not appear to be *T*. *gondii* strain type specific. In conclusion, tissue cysts in brain rarely reach a diameter of 70 μm in size, but there is variability in tissue cyst size between genotypes of *T*. *gondii* but no clear pattern. Tissue cysts were largest in rats infected with the atypical TgRabbitBr1 strain.

### Tissue cyst rupture and reactivation of latent infection

It is well known that *T*. *gondii* tissue cysts persist in organs of infected hosts for several months and perhaps for life, depending on the host and parasite strains [1]. Although more tissue cysts are found per gram of brain tissue in mice than in other hosts, data are limited to a few mouse-adapted strains and immunity to toxoplasmosis maybe dependent upon the strain of mouse and the parasite. Indeed tachyzoites were present in the brains of chronically infected mice [62] but the frequency of the phenomenen is not known. Parasitemias have been observed in chronically infected mice [17] and congenital infections have been documented in some strains of chronically infected mice [16,18]. Hermes et al. (2008) [63] reported neurological deficits in mice 11–16 months post infection with the ME49 strain of *T*. *gondii*. They found inflammatory lesions although no tachyzoites, few bradyzoites, and no inflammation were observed around intra neuronal tissue cysts. Ferguson et al. (1991) [27] found persistent subclinical meningoencephalitis in mice during the chronic phase (1–22 months p.i.).

*Toxoplasma gondii* tissue cyst rupture has been documented only rarely. In a quantitative study, tissue cyst rupture was found in only two (0.27%) of 750 tissue cysts examined in the brains of experimentally infected mice, although *T*. *gondii*-positive debris was found in eight (1.4%) glial nodules [27,64]. Recently, Watts et al. (2015) [31] quantitavely studied the dynamics of tissue cysts in CBA/J mice infected with the ME49 strain. Tissue cysts were separated by percoll gradient fractionation from brains of mice euthanized at 3, 4, 5, 6, and 8 weeks p.i. The number of tissue cysts and their sizes were remarkably similar 3–8 weeks p.i.; both small (<40 μm) and large (>70 μm) tissue cysts occurred at all time points. There was no evidence for cyclic rupture of tissue cysts and formation of many new tissue cysts. Evidence indicated that the loss of tissue cysts (if any) is balanced by emergence of an equal number of tissue cysts destroyed. Whether this phenomenon is peculiar to the strain of mouse and the ME49 strain requires further study [31].

Tissue cyst rupture was documented in a Panamanian night monkey (*Aotus lemurinus*) that had been inoculated three times with an attenuated (ts-4) strain of *T*. *gondii* and then reinoculated orally with tissue cysts of a wild type *T*. *gondii* strain [65,66]. Tissue cyst rupture has rarely been documented in naturally infected immunocompetent hosts. One of us (JPD), who has examined tissues of many naturally infected animals, found ruptured tissue cysts only twice, in a cat [67] and a kangaroo [68], and in both instances the parasite appeared to be morphologically different than *T*. *gondii*.

In the present study in experimentally infected rats, tissue cyst rupture was frequent. Similar observations have been made previously in rats orally inoculated with VEG strain oocysts [32]. However, there was no evidence for reactivation and the presence of tachyzoites. Although observations were limited to rats infected for two months, there was no evidence for the formation of new tissue cysts.

### Ocular toxoplasmosis

One of the most important consequences of toxoplasmosis is ocular disease. Toxoplasmosis can cause loss of vision, and even total blindness in humans [69,70]. Treatment of ocular toxoplasmosis is difficult because the currently available medicines do not kill tissue cysts, and drugs do not diffuse well into the retina, the site most commonly affected. Many animal models have been used to study ocular toxoplasmosis (reviewed in Su et al., 2014; Luder et al., 2014) [71,72] with observations in mice, hamsters, and rabbits inoculated parenterally with tachyzoites or intraocular injection. However, ocular toxoplasmosis has not been described in rats. Using the natural route (oral) and the stage often ingested (oocyst), lesions were found in eyes of 23 (25%) of 92 asymptomatic rats euthanized two months p.i. This prevalence is similar to that seen in human beings who have been infected during epidemics associated with ingestion of water contaminated with *T*. *gondii* oocysts [73,74]. This is particularly important as the search for ocular lesions was not exhaustive and hence higher infection rates are likely.

In human toxoplasmosis the most common site of clinical disease is the retina. Other tissue may be infected, but subclinically. Although there is inflammation in the choroid, *T*. *gondii* is not found in the choroid—the disease is correctly called retinochoroiditis [70]. It has been hypothesized that in humans, tissue cysts in retina can rupture causing inflammation with or without reactivation. Therefore, corticosteroids are sometimes used to reduce inflammation in conjunction with specific anti *T*. *gondii* therapy [73]. In the present study, eyes were affected early in infection as retinitis was found seven days p.i. and was associated with demonstrable tachyzoites, similar to ocular toxoplasmosis in humans [69,75]. Tissue cysts were found in the retina of 20 of 23 rats with lesions. In some eyes, tissue cysts were seen in retinal tissue without inflammation; the same phenomenon can be seen in human eyes. Parasites were found predominantly in the inner retina. The same has been reported with infection of human eyes, which has been attributed to their association with blood vessels, which are present in the layer [14,70,76]. The rat model might be useful to study pathogenesis of ocular toxoplasmosis.

## Supporting Information

S1 Fig Surface view of rat brain with slices 1 and 2 used for histological examination, and slice 3 homogenized in saline for tissue cyst measurements. (TIF)

S2 Fig Mid sagittal sections of both halves of brain of Rat D6223, from olfactory bulbs (a) to the cervical spinal cord (b). Tissue cysts are not visible at this magnification. PASH-stained. GT1 strain.
(TIF)

S1 Table Brain regions in rats with percentage of total area. (DOCX)

S2 Table Distribution of tissue cysts in different regions of the rat brain. (XLSX)

## References

[1] Dubey JP (2010) Toxoplasmosis of animals and humans. Boca Raton, FL: CRC Press 313 p.

[2] Grigg ME, Sundar N (2009) Sexual recombination punctuated by outbreaks and clonal expansions predicts *Toxoplasma gondii* population genetics. Int J Parasitol 39: 925–933. 10.1016/j.ijpara.2009.02.005 19217909 PMC2713429

[3] Vaudaux JD, Muccioli C, James ER, Silveira C, Magargal SL, Jung C, et al (2010) Identification of an atypical strain of *Toxoplasma gondii* as the cause of a waterborne outbreak of toxoplasmosis in Santa Isabel do Ivaí, Brazil. J Infect Dis 202: 1226–1233. 10.1086/656397 20836703 PMC5718918

[4] Elbez-Rubinstein A, Ajzenberg D, Dardé ML, Cohen R, Dumètre A, Yera H, et al (2009) Congenital toxoplasmosis and reinfection during pregnancy: case report, strain characterization, experimental model of reinfection, and review. J Infect Dis 199: 280–285. 10.1086/595793 19032062

[5] Ajzenberg D, Bañuls AL, Su C, Dumètre A, Demar M, Carme B, et al (2004) Genetic diversity, clonality and sexuality in *Toxoplasma gondii*. Int J Parasitol 34: 1185–1196. 15380690 10.1016/j.ijpara.2004.06.007

[6] Demar M, Ajzenberg D, Maubon D, Djossou F, Panchoe D, Punwasi W, et al (2007) Fatal outbreak of human toxoplasmosis along the Maroni River: epidemiological, clinical, and parasitological aspects. Clin Infect Dis 45: e88–e95. 17806043 10.1086/521246

[7] Sibley LD, Ajioka JW (2008) Population structure of *Toxoplasma gondii*: clonal expansion driven by infrequent recombination and selective sweeps. Ann Rev Microbiol 62: 329–351. 18544039 10.1146/annurev.micro.62.081307.162925

[8] Khan A, Dubey JP, Su C, Ajioka JW, Rosenthal BM, Sibley LD (2011) Genetic analyses of atypical *Toxoplasma gondii* strains reveals a fourth clonal lineage in North America. Int J Parasitol 41: 645–655. 10.1016/j.ijpara.2011.01.005 21320505 PMC3081397

[9] Shwab EK, Zhu XQ, Majumdar D, Pena HF, Gennari SM, Dubey JP, et al (2014) Geographical patterns of *Toxoplasma gondii* genetic diversity revealed by multilocus PCR-RFLP genotyping. Parasitology 141: 453–461. S0031182013001844 [pii]; 10.1017/S0031182013001844 24477076

[10] McLeod R, Boyer KM, Lee D, Mui E, Wroblewski K, Karrison T, et al (2012) Prematurity and severity are associated with Toxoplasma gondii alleles (NCCCTS, 1981–2009). Clin Infect Dis 54: 1595–1605. 10.1093/cid/cis258 22499837 PMC3348955

[11] Su C, Khan A, Zhou P, Majumdar D, Ajzenberg D, Dardé ML, et al (2012) Globally diverse *Toxoplasma gondii* isolates comprise six major clades originating from a small number of distinct ancestral lineages. Proc Natl Acad Sci USA 109: 5844–5849. 10.1073/pnas.1203190109 22431627 PMC3326454

[12] Di CM, Marocco D, Galizi R, Proietti C, Spaccapelo R, Crisanti A (2008) Temporal and spatial distribution of *Toxoplasma gondii* differentiation into Bradyzoites and tissue cyst formation in vivo. Infect Immun 76: 3491–3501. IAI.00254-08 [pii]; 10.1128/IAI.00254-08 18505811 PMC2493213

[13] Dubey JP, Frenkel JK (1976) Feline toxoplasmosis from acutely infected mice and the development of *Toxoplasma* cysts. J Protozool 23: 537–546. 1003342 10.1111/j.1550-7408.1976.tb03836.x

[14] Holland GN, Engstrom RE, Glasgow BJ, Berger BB, Daniels SA, Sidikaro Y, et al (1988) Ocular toxoplasmosis in patients with the acquired immunodeficiency syndrome. Am J Ophthalmol 106: 653–667. 3195645 10.1016/0002-9394(88)90697-6

[15] Remington JS, McLeod R, Thulliez P, Desmonts G (2006) Toxoplasmosis In: Remington JS, Klein JO, Wilson CB, Baker CJ, editors. Infectious diseases of the fetus and newborn infant. Philadelphia: Elsevier Saunders pp. 947–1091.

[16] Beverley JKA (1959) Congenital transmission of toxoplasmosis through successive generations of mice. Nature 183: 1348–1349. 13657122 10.1038/1831348a0

[17] Remington JS, Melton J, Jacobs L (1961) Induced and spontaneous recurrent parasitemia in chronic infections with avirulent strains of *Toxoplasma gondii*. J Immunol 87: 578–581. 14491398

[18] Remington JS, Jacobs L, Melton ML (1961) Congenital transmission of toxoplasmosis from mother animals with acute and chronic infections. J Infect Dis 108: 163–173. 13740759 10.1093/infdis/108.2.163

[19] Howe DK, Summers BC, Sibley LD (1996) Acute virulence in mice is associated with markers on chromosome VIII in *Toxoplasma gondii*. Infect Immun 64: 5193–5198. 8945565 10.1128/iai.64.12.5193-5198.1996 PMC174507

[20] Dubey JP, Frenkel JK (1998) Toxoplasmosis of rats: a review, with considerations of their value as an animal model and their possible role in epidemiology. Vet Parasitol 77: 1–32. 9652380 10.1016/s0304-4017(97)00227-6

[21] Vyas A, Kim SK, Giacomini N, Boothroyd JC, Sapolsky RM (2007) Behavioral changes induced by *Toxoplasma* infection of rodents are highly specific to aversion of cat odors. Proc Natl Acad Sci USA 104: 6442–6447. 17404235 10.1073/pnas.0608310104 PMC1851063

[22] Gonzalez LE, Rojnik B, Urrea F, Urdaneta H, Petrosino P, Colasante C, et al (2007) *Toxoplasma gondii* infection lower anxiety as measured in the plus-maze and social interaction tests in rats. A behavioral analysis. Behavioral Brain Research 177: 70–79. 10.1016/j.bbr.2006.11.012 17169442

[23] Berenreiterova M, Flegr J, Kubena AA, Nemec P (2011) The distribution of *Toxoplasma gondii* cysts in the brain of a mouse with latent toxoplasmosis: implications for the behavioral manipulation hypothesis. PLoS ONE 6: e28925 10.1371/journal.pone.0028925 22194951 PMC3237564

[24] Afonso C, Paixão VB, Costa RM (2012) Chronic *Toxoplasma* infection modifies the structure and the risk of host behavior. PLoS ONE 7: e32489 10.1371/journal.pone.0032489 22431975 PMC3303785

[25] Gatkowska J, Wieczorek M, Dziadek B, Dzitko K, Dlugonska H (2012) Behavioral changes in mice caused by *Toxoplasma gondii* invasion of brain. Parasitol Res 111: 53–58. 10.1007/s00436-011-2800-y 22223035 PMC3378833

[26] Sullivan AM, Zhao X, Suzuki Y, Ochiai E, Crutcher S, Gilchrist MA (2013) Evidence for finely-regulated asynchronous growth of *Toxoplasma gondii* cysts based on data-driven model selection. PLoS Comput Biol 9: e1003283 10.1371/journal.pcbi.1003283; PCOMPBIOL-D-13-00456 [pii]. 24244117 PMC3828147

[27] Ferguson DJP, Graham DI, Hutchison WM (1991) Pathological changes in the brains of mice infected with *Toxoplasma gondii*: a histological, immunocytochemical and ultrastructural study. Int J Exp Pathol 72: 463–474. 1883744 PMC2001958

[28] Melzer TC, Cranston HJ, Weiss LM, Halonen SK (2010) Host cell preference of *Toxoplasma gondii* cysts in murine brain: a confocal study. J Neuroparasitology 1. 10.4303/jnp/N100505 PMC3103221 21625284

[29] Ferguson DJP, Hutchison WM (1987) An ultrastructural study of the early development and tissue cyst formation of *Toxoplasma gondii* in the brains of mice. Parasitol Res 73: 483–491. 3422976 10.1007/BF00535321

[30] van der Waaij D (1959) Formation, growth and multiplication of *Toxoplasma gondii* cysts in mouse brains. Trop Geogr Med 11: 345–360.

[31] Watts E, Zhao Y, Dhara A, Eller B, Patwardhan A, Sinai AP (2015) Novel approaches reveal that *Toxoplasma gondii* bradyzoites within tissue cysts are dynamic and replicating entities in vivo. mBio 6: e01155–15. mBio.01155-15 [pii]; 10.1128/mBio.01155-15 26350965 PMC4600105

[32] Dubey JP (1996) Pathogenicity and infectivity of *Toxoplasma gondii* oocysts for rats. J Parasitol 82: 951–956. 8973405

[33] Berdoy M, Webster JP, MacDonald DW (2000) Fatal attraction in rats infected with *Toxoplasma gondii*. Proc R Soc Lond B 267: 1591–1594. 10.1098/rspb.2000.1182 PMC1690701 11007336

[34] Webster JP (2001) Rats, cats, people and parasites: the impact of latent toxoplasmosis on behaviour. Microbes Infect 3: 1037–1045. 11580990 10.1016/s1286-4579(01)01459-9

[35] Lamberton PHL, Donnelly CA, Webster JP (2008) Specificity of the *Toxoplasma gondii*-altered behaviour to definitive versus non-definitive host predation risk. Parasitology 135: 1143–1150. 10.1017/S0031182008004666 18620624

[36] Webster JP, Lamberton PH, Donnelly CA, Torrey EF (2006) Parasites as causative agents of human affective disorders? The impact of anti-psychotic, mood-stabilizer and anti-parasite medication on *Toxoplasma gondii*'s ability to alter host behaviour. Proc Biol Sci 273: 1023–1030. F314V9210P112451 [pii]; 10.1098/rspb.2005.3413 16627289 PMC1560245

[37] Martin HL, Alsaady I, Howell G, Prandovszky E, Peers C, Robinson P, et al (2015) Effect of parasitic infection on dopamine biosynthesis in dopaminergic cells. Neuroscience 306: 50–62. S0306-4522(15)00728-9 [pii]; 10.1016/j.neuroscience.2015.08.005 26297895 PMC4577654

[38] Prandovszky E, Gaskell E, Martin H, Dubey JP, Webster JP, McConkey GA (2011) The neurotropic parasite *Toxoplasma gondii* increases dopamine metabolism. PLoS ONE 6: e23866 10.1371/journal.pone.0023866 21957440 PMC3177840

[39] Skallova A, Kodym P, Frynta D, Flegr J (2006) The role of dopamine in *Toxoplasma*-induced behavioural alterations in mice: an ethological and ethopharmacological study. Parasitology 133: 525–535. 16882355 10.1017/S0031182006000886

[40] Brown AS, Derkits EJ (2010) Prenatal infection and schizophrenia: a review of epidemiologic and translational studies. Am J Psychiatry 167: 261–280. appi.ajp.2009.09030361 [pii]; 10.1176/appi.ajp.2009.09030361 20123911 PMC3652286

[41] Torrey EF, Yolken RH (2007) Schizophrenia and toxoplasmosis. Schizophr Bull 33: 727–728. sbm026 [pii]; 10.1093/schbul/sbm026 17426051 PMC2526129

[42] Torrey EF, Bartko JJ, Lun ZR, Yolken RH (2007) Antibodies to *Toxoplasma gondii* in patients with schizophrenia: a meta-analysis. Schizophr Bull 33: 729–736. sbl050 [pii]; 10.1093/schbul/sbl050 17085743 PMC2526143

[43] Dubey JP (1980) Mouse pathogenicity of *Toxoplasma gondii* isolated from a goat. Am J Vet Res 41: 427–429. 7369619

[44] Dubey JP, Su C, Cortés JA, Sundar N, Gomez-Marin JE, Polo LJ, et al (2006) Prevalence of *Toxoplasma gondii* in cats from Colombia, South America and genetic characterization of *T*. *gondii* isolates. Vet Parasitol 141: 42–47. 16797845 10.1016/j.vetpar.2006.04.037

[45] Lunde MN, Jacobs L (1983) Antigenic differences between endozoites and cystozoites of *Toxoplasma gondii*. J Parasitol 69: 806–808. 6200590

[46] Dubey JP, Passos LMF, Rajendran C, Ferreira LR, Gennari SM, Su C (2011) Isolation of viable *Toxoplasma gondii* from guinea fowl (*Numida meleagris*) and domestic rabbits (*Oryctolagus cuniculus*) from Brazil. J Parasitol 97: 842–845. 10.1645/GE-2728.1 21506805

[47] Parmley SF, Gross U, Sucharczuk A, Windeck T, Sgarlato GD, Remington JS (1994) Two alleles of the gene encoding surface antigen P22 in 25 strains of *Toxoplasma gondii*. J Parasitol 80: 293–301. 7908967

[48] Dubey JP, Lunney JK, Shen SK, Kwok OCH, Ashford DA, Thulliez P (1996) Infectivity of low numbers of *Toxoplasma gondii* oocysts to pigs. J Parasitol 82: 438–443. 8636849

[49] Dubey JP, Rajendran C, Ferreira LR, Martins J, Kwok OCH, Hill DE, et al (2011) High prevalence and genotypes of *Toxoplasma gondii* isolated from goats, from a retail meat store, destined for human consumption in the USA. Int J Parasitol 41: 827–833. 10.1016/j.ijpara.2011.03.006 21515278

[50] Dubey JP, Rajendran C, Ferreira LR, Kwok OCH, Sinnett D, Majumdar D, et al (2010) A new atypical highly mouse virulent *Toxoplasma gondii* genotype isolated from a wild black bear in Alaska. J Parasitol 96: 713–716. 10.1645/GE-2429.1 20486739

[51] Dubey JP, Thulliez P, Powell EC (1995) *Toxoplasma gondii* in Iowa sows: comparison of antibody titers to isolation of *T*. *gondii* by bioassays in mice and cats. J Parasitol 81: 48–53. 7876977

[52] Velmurugan GV, Su C, Dubey JP (2009) Isolate designation and characterization of *Toxoplasma gondii* isolates from pigs in the United States. J Parasitol 95: 95–99. 10.1645/GE-1746.1 19245283

[53] Dubey JP, Zhu XQ, Sundar N, Zhang H, Kwok OCH, Su C (2007) Genetic and biologic characterization of *Toxoplasma gondii* isolates of cats from China. Vet Parasitol 145: 352–356. 17267132 10.1016/j.vetpar.2006.12.016

[54] Dubey JP (1992) Isolation of *Toxoplasma gondii* from a naturally infected beef cow. J Parasitol 78: 151–153. 1738059

[55] Dubey JP (1995) Duration of immunity to shedding of *Toxoplasma gondii* oocysts by cats. J Parasitol 81: 410–415. 7776126

[56] McAllister MM, Parmley SF, Weiss LM, Welch VJ, McGuire AM (1996) An immunohistochemical method for detecting bradyzoite antigen (BAG5) in *Toxoplasma gondii*-infected tissues cross-reacts with a *Neospora caninum* bradyzoite antigen. J Parasitol 82: 354–355. 8604117

[57] Paxinos G, Watson C (2006) The Rat Brain in Stereotaxic Coordinates. Academic Press. 10.1016/0165-0270(80)90021-7 6110810

[58] Dubey JP, Desmonts G (1987) Serological responses of equids fed *Toxoplasma gondii* oocysts. Equine Vet J 19: 337–339. 3622463 10.1111/j.2042-3306.1987.tb01426.x

[59] McConkey GA, Martin HL, Bristow GC, Webster JP (2013) *Toxoplasma gondii* infection and behaviour—location, location, location? J Exp Biol 216: 113–119. 216/1/113 [pii]; 10.1242/jeb.074153 23225873 PMC3515035

[60] Dubey JP, Lindsay DS, Speer CA (1998) Structure of *Toxoplasma gondii* tachyzoites, bradyzoites and sporozoites, and biology and development of tissue cysts. Clin Microbiol Rev 11: 267–299. 9564564 10.1128/cmr.11.2.267 PMC106833

[61] Beverley JKA (1958) A rational approach to the treatment of toxoplasmic uveitis. Trans Ophthalmol Soc U K 78: 109–121. 13635727

[62] Dubey JP, Speer CA, Shen SK, Kwok OCH, Blixt JA (1997) Oocyst-induced murine toxoplasmosis: life cycle, pathogenicity, and stage conversion in mice fed *Toxoplasma gondii* oocysts. J Parasitol 83: 870–882. 9379292

[63] Hermes G, Ajioka JW, Kelly KA, Mui E, Roberts F, Kasza K, et al (2008) Neurological and behavioral abnormalities, ventricular dilatation, altered cellular functions, inflammation, and neuronal injury in brains of mice due to common, persistent, parasitic infection. J Neuroinflammation 5: 48 1742-2094-5-48 [pii]; 10.1186/1742-2094-5-48 18947414 PMC2588578

[64] Ferguson DJP, Hutchison WM, Pettersen E (1989) Tissue cyst rupture in mice chronically infected with *Toxoplasma gondii*. An immunocytochemical and ultrastructural study. Parasitol Res 75: 599–603. 2771928 10.1007/BF00930955

[65] Frenkel JK, Escajadillo A (1987) Cyst rupture as a pathogenic mechanism of toxoplasmic encephalitis. Am J Trop Med Hyg 36: 517–522. 3578650 10.4269/ajtmh.1987.36.517

[66] Escajadillo A, Frenkel JK (1991) Experimental toxoplasmosis and vaccine tests in Aotus monkeys. Am J Trop Med Hyg 44: 382–389. 2042705 10.4269/ajtmh.1991.44.382

[67] Dubey JP, Fenner WR (1993) Clinical segmental myelitis associated with an unidentified *Toxoplasma*-like parasite in a cat. J Vet Diagn Invest 5: 472–480. 8373868 10.1177/104063879300500334

[68] Dubey JP, Ott-Joslin J, Torgerson RW, Topper MJ, Sundberg JP (1988) Toxoplasmosis in black-faced kangaroos (*Macropus fuliginosus melanops*). Vet Parasitol 30: 97–105. 3245110 10.1016/0304-4017(88)90156-2

[69] Arantes TE, Silveira C, Holland GN, Muccioli C, Yu F, Jones JL, et al (2015) Ocular involvement following postnatally acquired *Toxoplasma gondii* infection in southern brazil: a 28-year experience. Am J Ophthalmol 159: 1002–1012. S0002-9394(15)00101-4 [pii]; 10.1016/j.ajo.2015.02.015 25743338

[70] Holland GN, O'Connor GR, Belfort R Jr, Remington JS (1996) Toxoplasmosis In: Pepose JS, Holland GS, Wilhelmus KR, editors. Ocular Infection and Immunity. St. Louis: Mosby pp. 1183–1223.

[71] Su E, Honda A, Latkany P (2014) Ocular disease due to *Toxoplasma gondii* In: Weiss LM, Kim K, editors. *Toxoplasma gondii*, the model Apicomplexan: Perspectives and methods. London: Academic Press pp. 161–192.

[72] Lüder CGK, Reichard U, Gross U (2014) *Toxoplasma* animal models and therapeutics In: Weiss LM, Kim K, editors. *Toxoplasma gondii*, the model Apicomplexan: Perspectives and methods. London: Academic Press pp. 217–255.

[73] Silveira C, Muccioli C, Holland GN, Jones JL, Yu F, de PA, et al (2015) Ocular Involvement Following an Epidemic of *Toxoplasma gondii* Infection in Santa Isabel do Ivai, Brazil. Am J Ophthalmol 159: 1013–1021. S0002-9394(15)00103-8 [pii]; 25743340 10.1016/j.ajo.2015.02.017

[74] Burnett AJ, Shortt SG, Isaac-Renton J, King A, Werker D, Bowie WR (1998) Multiple cases of acquired toxoplasmosis retinitis presenting in an outbreak. Ophthalmology 105: 1032–1037. 9627653 10.1016/S0161-6420(98)96004-3

[75] Holland GN, Muccioli C, Silveira C, Weisz JM, Belfort R Jr, O'Connor GR (1999) Intraocular inflammatory reactions without focal necrotizing retinochoroiditis in patients with acquired systemic toxoplasmosis. Am J Ophthalmol 128: 413–420. 10577581 10.1016/s0002-9394(99)00300-1

[76] Nicholson DH, Wolchok EB (1976) Ocular toxoplasmosis in an adult receiving long-term corticosteroid therapy. Arch Ophthalmol 94: 248–254. 1252177 10.1001/archopht.1976.03910030120009

