# Supplementary figures and images for: Experimental Toxoplasmosis in Rats Induced Orally with Eleven Strains of Toxoplasma gondii of Seven Genotypes: Tissue Tropism, Tissue Cyst Size, Neural Lesions, Tissue Cyst Rupture without Reactivation, and Ocular Lesions
Source: PLoS One. 2016 May 26;11(5):e0156255. doi: 10.1371/journal.pone.0156255 (PMC4882154; doi:10.1371/journal.pone.0156255)

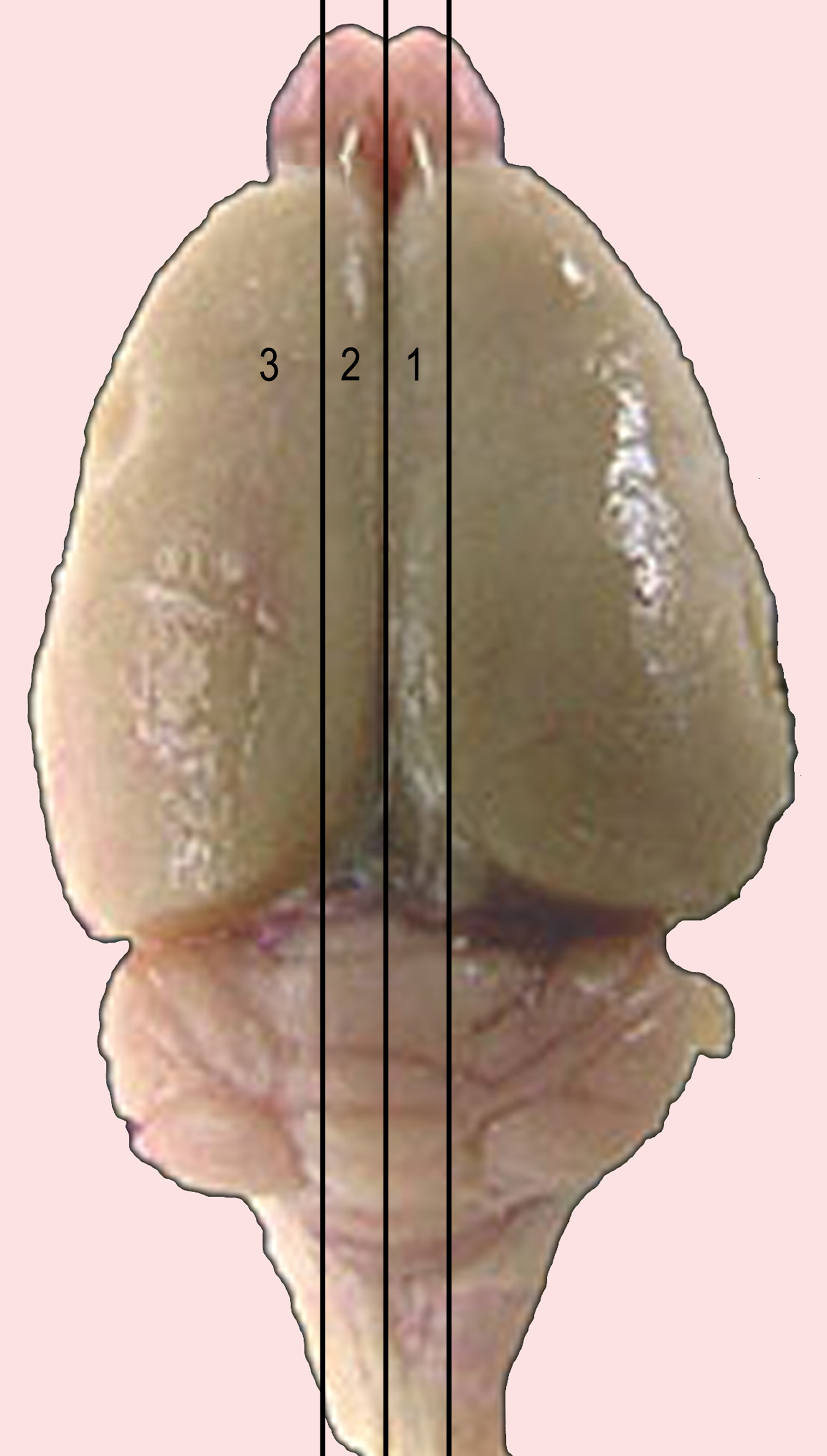

Supplement: S1 Fig — (TIF) [file pone.0156255.s001.tif]

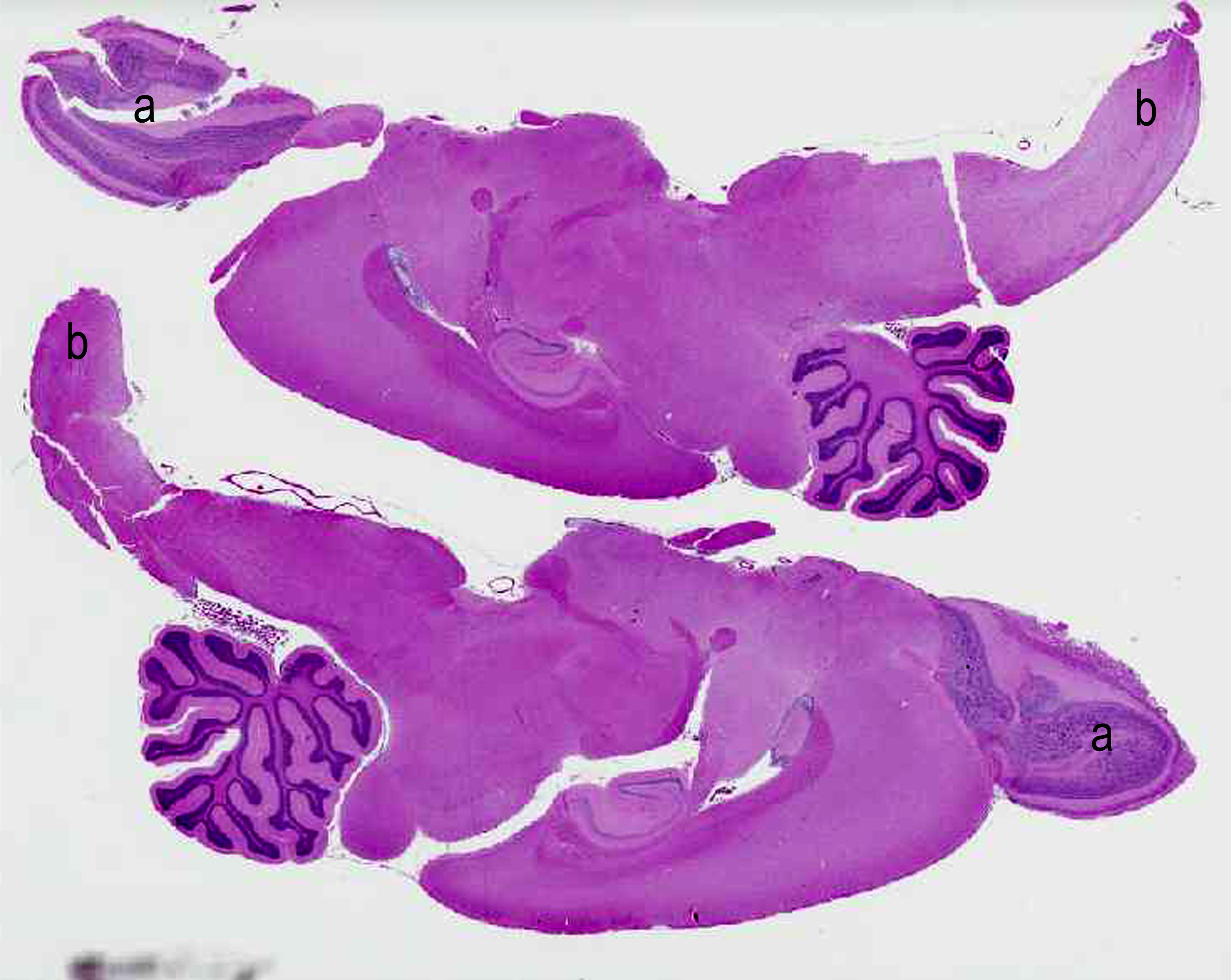

Supplement: S2 Fig — Tissue cysts are not visible at this magnification. PASH-stained. GT1 strain. (TIF) [file pone.0156255.s002.tif]
