# Supplementary material for: Experimental Toxoplasmosis in Rats Induced Orally with Eleven Strains of Toxoplasma gondii of Seven Genotypes: Tissue Tropism, Tissue Cyst Size, Neural Lesions, Tissue Cyst Rupture without Reactivation, and Ocular Lesions
Source: PLoS One. 2016 May 26;11(5):e0156255. doi: 10.1371/journal.pone.0156255 (PMC4882154; doi:10.1371/journal.pone.0156255)
Supplement: S1 Table — (DOCX) [file pone.0156255.s003.docx]

**Table 1S**. **Brain regions in rats with percentage of total area**.

| Region | Percentage |
| --- | --- |
| Cerebellum | 15 |
| Cerebral Cortex | 23 |
| Colliculus | 4 |
| Hippocampus | 3 |
| Hypothalamus | 8 |
| Mecencephalon | 7 |
| Olfactory Bulb | 7 |
| Rhombencephalon | 15 |
| Subpallium | 9 |
| Thalamus | 9 |
| Total | **100** |
